# Industrial decarbonization under Japan’s national mitigation scenarios: a multi-model analysis

**DOI:** 10.1007/s11625-021-00905-2

**Published:** 2021-02-16

**Authors:** Yiyi Ju, Masahiro Sugiyama, Etsushi Kato, Yuhji Matsuo, Ken Oshiro, Diego Silva Herran

**Affiliations:** 1 Institute for Future Initiatives, University of Tokyo, Tokyo, 113-0033 Japan; 2 Institute of Applied Energy, Tokyo, Japan; 3 Institute of Energy Economics, Tokyo, Japan; 4 Department of Environmental Engineering, Kyoto University, Kyoto, Japan; 5 Institute for Global Environmental Strategies, Kanagawa, Japan; 6 National Institute for Environmental Studies, Tsukuba, Japan

**Keywords:** Industry, Model intercomparison project, Nationally determined contribution, Japan

## Abstract

Energy-intensive industries are difficult to decarbonize. They present a major challenge to the emerging countries that are currently in the midst of rapid industrialization and urbanization. This is also applicable to Japan, a developed economy, which retains a large presence in heavy industries compared to other developed economies. In this paper, the results obtained from four energy-economic and integrated assessment models were utilized to explore climate mitigation scenarios of Japan’s industries by 2050. The results reveal that: (i) Japan’s share of emissions from industries may increase by 2050, highlighting the difficulties in achieving industrial decarbonization under the prevailing industrial policies; (ii) the emission reduction in steelmaking will play a key role, which can be achieved by the implementation of carbon capture and expansion of hydrogen technologies after 2040; (iii) even under mitigation scenarios, electrification and the use of biomass use in Japan’s industries will continue to be limited in 2050, suggesting a low possibility of large-scale fuel switching or end-use decarbonization. After stocktaking of the current industry-sector modeling in integrated assessment models, we found that such limited uptake of cleaner fuels in the results may be related to the limited interests of both participating models and industry stakeholders in Japan, specifically the interests on the technologies that are still at the early stage of development but with high reduction potential. It is crucial to upgrade research and development activities to enable future industry-sector mitigation as well as to improve modeling capabilities of energy end-use technologies in integrated assessment models.

**Supplementary Information:**

The online version contains supplementary material available at 10.1007/s11625-021-00905-2.

## Introduction

The 25^th^ Conference of the Parties (COP25) to the United Nations Framework Convention on Climate Change reiterated the need for urgent action on climate change, stating the need for more efforts to achieve climate goals in order to stabilize the global temperature rise at 1.5 °C by the end of the century (IPCC 2018). On the other hand, the bottom-up approach of the Paris Agreement implied that policies should be developed based on the careful assessment of the unique situation of each country. For Japan, which prides itself on *monozukuri* (manufacturing) and retains a high share in heavy industries (METI 2020), this means that policies must address long-term decarbonization of the indsutry sector. The importance of industrial decarbonization has been mentioned in our previous paper (Sugiyama et al. 2019).

**Fig. 1:**
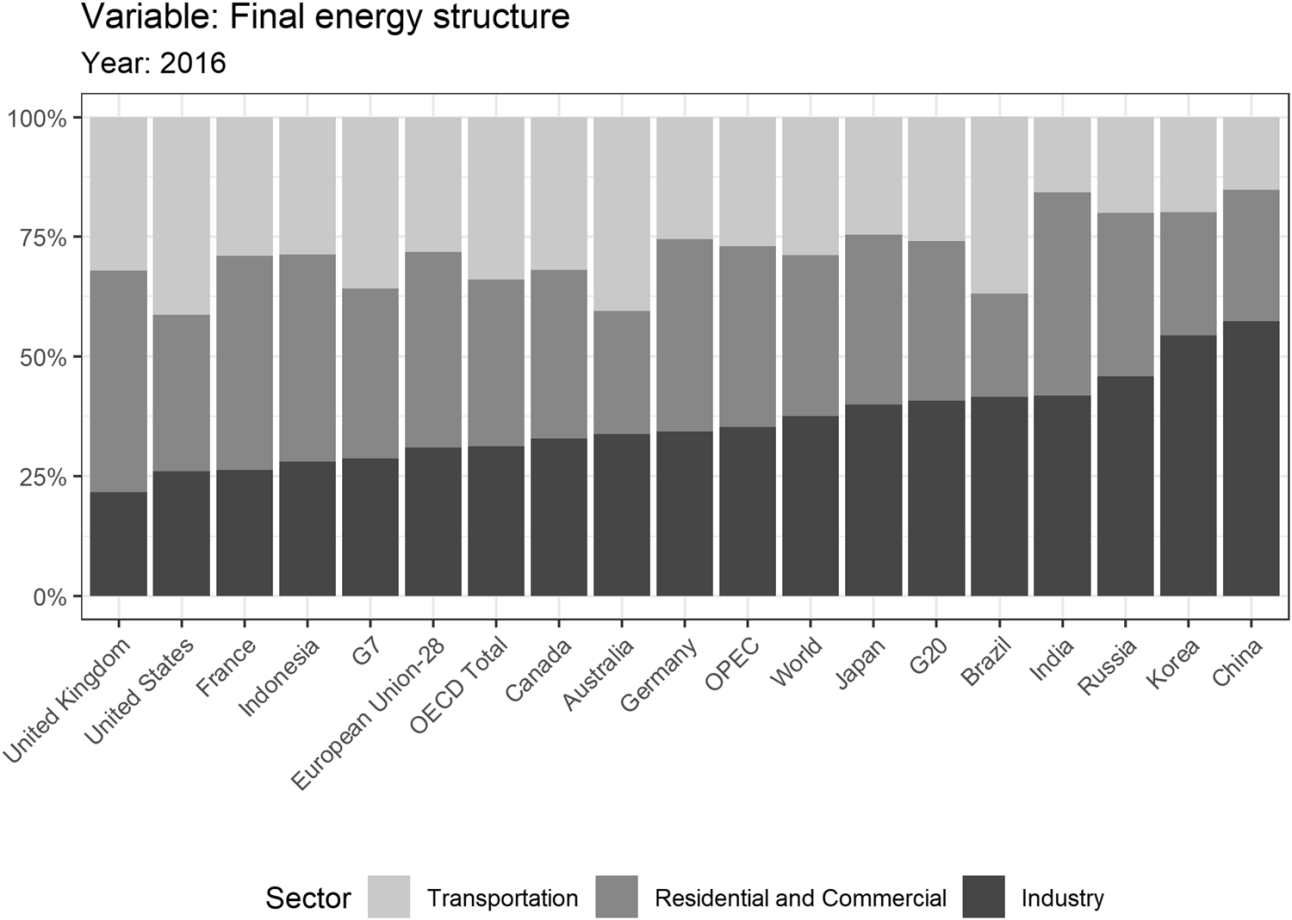
Sectoral shares of final energy consumption in different countries/regions. Source: summarized from IEA (2016), sorted according to the share of the industry sector

Energy-intensive industries, such as steel and cement sectors, are extremely difficult to decarbonize in the short run due to the increasing demand for industrial products and subsidies from national strategies (Åhman et al. 2017), the time taken to update energy infrastructure (Davis et al. 2018), and the existence of process emissions (besides those from fuel combustion) and the need for high-temperatures. This is particularly true for emerging countries that are in the midst of rapid industrialization and urbanization, such as China, India, and Brazil (Fig. 1).

Although Japan is a member of the Group of Seven (G7), the share of its industries in comparison with its total final energy consumption is much higher than the G7 average. In fact, it is closer to the average of the Group of Twenty (G20), which includes emerging economies.

The Government of Japan has taken numerous steps to promote mitigation in the industry sector. The Plan for Global Warming Countermeasure (GoJ 2016) and the Intended Nationally Determined Contribution submitted to United Nations Framework Convention on Climate Change (GoJ 2015) acknowledged the contribution of industries to emission reduction since 2013 and has called for continued efforts. The underlying principle is that climate change mitigation should not harm economic growth, but it should simultaneously contribute to the achievement of other policy goals, such as economy, productivity, and added value growth (Long-Term Low-Carbon Vision, MOE 2016a; also Long-Term Growth Strategy based on the Paris Agreement: Cabinet Decision, MOE 2019a). This approach has mostly relied on voluntary action, especially of the Japan Business Federation (JBF; Keidanren), and actions driven by Energy Conservation Law to improve energy efficiency.

Keidanren (Japan Business Federation) formulated its first voluntary action plan in 1998 termed Voluntary Action Plan for the Environment. The aim of this plan was to focus on climate change mitigation after the Kyoto Protocol agreement in 1997. The plan covered 38 industries, including energy-intensive sectors, such as steel, cement, and machinery. After the first commitment period of the Kyoto Protocol, it was renamed The Action Plan for the Low Carbon Society. Since 2008, evaluation of emission reduction has been conducted annually by a government committee and a third-party committee. In the 2014 evaluation report (JBF 2014), it was reported that JBF members contributed over 80% to the total domestic industrial emissions and achieved a 5.6% reduction in emissions, as compared to the 2005 level.

The Act on the Rational Use of Energy, also known as the Energy Conservation Law, was enacted in Japan in 1979 and was upgraded several times in order to respond to social needs. It directly covered entities from the industry and transport sector and promoted an efficient energy management system. The obligation of entities included a periodic report on energy consumption, implementation of specified measures in the guidelines (adjustment of operating hours), and implementation of energy conservation measures (METI 2013).

Another major feature of the Act was to set energy efficiency standards for various types of products, including appliances and vehicles. Accordingly, the Top Runner Program was executed, wherein standards were set according to the level of the best performing products (top-runners) in the past years (METI 2015a). 31 products, including passenger vehicles and air-conditioners have been covered under this program as of 2020.

Of the various mitigation approaches, energy efficiency has been the main priority, which has made Japan one of the most energy efficient economies. The converse is that since there is a decreasing return to energy efficiency investments, Japan now has a limited, domestic energy conservation potential (IEA 2016; Kuramochi 2016). It, therefore, pushed for international mechanisms, such as the Joint Crediting Mechanism (and the Clean Development Mechanism). In this, if a partner country installs an efficient, Japanese technology, emission reduction against the baseline is counted as a credit. Furthermore, the Ministry of Environment also stated Japan’s financial and technical contributions to developing countries at the COP20 (MOE 2014). Moreover, Japan is promoting inter-industry and international cooperation (MOE 2016b; MOE 2019b; METI 2019a).

Besides energy efficiency, other mitigation measures have been explored for industries. Such measures include Carbon Capture and Storage (CCS), Carbon Capture, Utilization, and Storage (CCUS; MOE 2008), introduction of renewables into industrial production processes (JISF 2014), and the application and development of low-carbon products and infrastructure (JBF 2019). For CCS, few demonstration projects have been conducted. Recently, the Tomakomai project was completed with an injection of 300,000 t-CO_2_ (METI 2019b; IEA 2017a). For the introduction of hydrogen, the COURSE50 project has been developed and is expected to reduce 30% of CO_2_ emissions in steelmaking industries (JISF 2014).

Some price instruments are applicable to the industry sector too, though they tax by fossil fuel type and the stringency is weak (exemptions and refunds in certain raw material industries, MOE 2014). Emission trading schemes (Tokyo market and Saitama market) started to work in force after 2010. It mainly targeted buildings and but also covered 580 factories as liable entities (ICAP 2020a,2020b). However, these were limited to only 2 out of the 47 prefectures of Japan.

Recent studies have identified new opportunities for industrial mitigation. The proposed approaches include improving material efficiency (Hertwich et al. 2019; UNIDO 2018; Grubler et al. 2018), negative emissions technologies (IEA 2019; ICEF 2016), bridging technology gaps (UNIDO 2016; Bataille et al. 2018), and increasing the uptake of renewables in industries (IEA 2017b; McMillan et al. 2016). There is also interest in digitalization, such as artificial intelligence and internet of things. In Japan, the concept of Society 5.0 is used to describe a new, human-centric digital society (METI 2017; MOE 2016b).

However, unlike the emphasis of policies and actions on the industry sector, in the model community, it seems that models are slow to include a detailed representation of industries compared to the transport sector (Sugiyama et al. 2014). Among the 21 models that contributed to the IPCC’s report titled Global warming of 1.5 °C (IPCC 2018), an endogenous and explicit representation of the electrification of transport demand (e.g., electric vehicles, electric rail) is observed in 17 models, while only 9 of the 21 models focus on the electrification of industrial energy demand (e.g., electric arc furnace, heat pumps, electric boilers, conveyor belts, extensive use of motor control, induction heating, and industrial use of microwave heating).

Meanwhile, previous analysis from a multi-model study targeting Japan shows that the large-scale deployment of low-carbon energy (such as nuclear, renewable, and carbon capture and storage) in the energy supply side is shared across most of the 9 participating models in scenarios consistent with 1.5 or 2 degrees of global warming (Oshiro et al. 2019). Improving the value-added of industrial products is also suggested in a study proposing a roadmap towards a low-carbon society in Japan (Ashina et al. 2012). In addition to such technology deployment in the energy supply side and expectation of industrial structure changes, previous studies also paid special attention to the diffusion of energy-efficient technologies in industries (Akashi 2012; Oda et al. 2007). However, such review summaries are scarce. To fill in such a gap, as the first multi-model analysis of industries in Japan, this paper further investigates the industry-related emissions under different sets of climate policy, energy demand, and technology scenarios, which contributes to a better understanding of industrial decarbonization in Japan.

Given the different emphasis on mitigation measures by different policies, namely energy saving, CCS, lower demand, and energy end-use technology changes, the aim of this paper was to answer the following research questions:

- How high would industrial energy consumption and emissions go by 2050? How does it compare to other sectors, other historical periods of Japan, or reports from other model teams?
- What are the most important mitigation measures for the industry sector and its sub-sectors?
  - Can industrial decarbonization solely count on energy saving? Does the industry sector in Japan need CCS? How well does low demand work? Will there be an increase in the uptake of clean energy carriers (electricity, biomass, and hydrogen) in industries in the future?

In addition, we ask the following modeling question:

- What is the status of industry-sector modeling in Japanese energy-economic and integrated assessment models? What should be expanded?

In this paper, the data from four energy-economic and integrated assessment models, AIM/Hub-Japan, AIM/Enduse-Japan, IEEJ_Japan 2017, and TIMES-Japan, were utilized to analyze the future scenarios of Japan’s industry by 2050, followed by a decomposition of emission changes based on the Kaya identity to investigate how Japan’s industrial decarbonization would be driven. Based on our previous work (Suguyama et al. 2019), this paper stock-takes of the current industry-sector modeling also helps to clarify the modeling status that is underway as well as the potential improvements in the modeling of end-use technologies in industries.

## Methodology

### Participating models

The multi-model analysis is based on the Stanford Energy Modeling Forum (EMF) 35 Japan Model Intercomparison Project (JMIP). The participating models include AIM/Hub-Japan, AIM/Enduse-Japan, IEEJ_Japan 2017, and TIMES-Japan, wherein AIM/Hub-Japan is a general equilibrium (GE) model and the rest are partial equilibrium (PE) models.

The GE model AIM/Hub-Japan has price-elastic service demands, while other partial equilibrium models follow exogenous service demands. Considering such differences, the GE model is separated from the group of PE models in part of the following results. Other differences among models, such as the industrial energy coverage and its data, as well as the industrial emissions coverage and its data source, are listed in Table ESM i.

### Scenario design

The scenario design of EMF35 JMIP considers four dimensions: policy, technology, demand, and imports. A description of all these scenarios are listed in Sugiyama et al. (2021, this issue). This paper focuses on the following scenarios:

- Base_Def: the baseline scenario, left to the individual modeling group's choice, with no additional climate policies and no other sub-regional emission reduction targets.
- 26by30 + 80by50_Def: the NDC&MCS scenario, where the models apply Japan’s Nationally Determined Contribution (NDC, 26% emissions reduction by FY2030 relative to the FY2013 levels) and Mid-Century Strategy (MCS, 80% emissions reduction by 2050).
- 26by30 + 80by50_NoCCS: same as the NDC&MCS scenario but without CCS deployment. CCS is considered as a key mitigation technology in the industry sector (ICEF 2016; Kuramochi et al. 2012). This scenario intends to look at the impact of unavailability of this technology.
- 26by30 + 80by50_LoDem: same as the NDC&MCS scenario but with lower growth in GDP per-capita, based on SSP2. Research institutes in Japan generally assume lower expectations in GDP per-capita growth (Kuriyama et al. 2019), compared to the 1.7% per year growth from 2015 to 2030 that is assumed by Japan’s NDC and MCS (METI 2015b).
- 26by30 + 80by50_LoDemInd: This scenario assumes that the energy service demand in the industry sector will be further reduced by 50% by 2050. As the industry sector is identified as an important sector (Sugiyama et al. 2019), reducing its service demand (Fujimori et al. 2014) may decrease the policy costs. The “50%” value underlines a range of possibilities that may lower the energy service demands in the future. Such a drop may happen intentionally due to improvements in material efficiency or final consumption preferences shifting towards smart devices and low-carbon products. It can also occur unintentionally because of natural disasters, global financial crises, pandemics, and similar extreme events (McCollum et al. 2020). In fact, in May 2020, the largest steelmaker in Japan, that is Nippon Steel, cut down 30% of its capacity partly due to the COVID-19, which is almost the same level of capacity cuts during the steel recession after 1985 Plaza agreement (Nikkei 2020).

Population growth in all models follows the same assumption (the middle population projection by the National Institute of Population and Social Security Research, IPSS, 2017) under all scenarios.

These four scenarios are selected from the whole set of scenarios in the EMF35 JMIP study as they cover almost the entire range of results, at least with respect to the total final energy consumption of the industry sector and its energy-related CO_2_ emissions (see Fig. ESM i).

By looking at the variables under all the selected scenarios (e.g., CO_2_ emissions from energy consumption of industries, final energy consumption of electricity by industries), the contribution of several important mitigation measures in the industry sector can be revealed. For example, the impacts of CCS can be shown by comparing results under 26by30 + 80by50_Def and 26by30 + 80by50_NoCCS. The impacts of lower demands can be shown by comparing results under 26by30 + 80by50_Def and 26by30 + 80by50_LoDem/LoDemInd. Moreover, a further Kaya decomposition can show the contribution of the improvements in energy efficiency (by energy intensity factor), the energy end-use technology changes and industrial electrification (by energy intensity factor).

### Decomposition of emission changes based on the Kaya identity

In this paper, decomposition of emission changes is conducted based on the Kaya identity (Ehrlich and Holdren 1971; Kaya 1990; Yamaji et al. 1991). The CO_2_ emissions in each sub-sector are decomposed into four factors, namely population, per-capita production (production of the final product in that sub-sector), energy intensity, and emission intensity (Eq. 1):

1 $${\text{EMS}} = {\text{POP}} \cdot \frac{{{\text{PRD}}}}{{{\text{POP}}}} \cdot \frac{{{\text{ENE}}}}{{{\text{PRD}}}} \cdot \frac{{{\text{EMS}}}}{{{\text{ENE}}}} = p \cdot d \cdot e \cdot i,$$

where $\mathrm{EMS}$ represents the CO_2_ emissions in each sub-sector, $\mathrm{POP}$ represents the national population, $\mathrm{PRD}$ represents the production in each sub-sector, and $\mathrm{ENE}$ represents the final energy consumption in each sub-sector. Correspondingly, $p$ represents population, $d$ represents per-capita production, $e$ represents energy intensity of production, and $i$ represents emission intensity.

Decomposition with $n$ factors has $n!$ unique decompositions; moreover, there are numerous ways to deal with non-uniqueness. The refined Laspeyers decomposition (Ang 2000; Peters et al, 2017) was utilized to ensure that the decomposition results had no residuals and that it satisfied the characteristics of time reversal, factor reversal, and zero-value robustness. The change in CO_2_ emissions between year $t$ and year $0$ can be decomposed to

2 $$\begin{aligned} & \frac{{\Delta {\text{EMS}}}}{{{\text{EMS}}_{0} }} = \frac{{{\text{EMS}}_{t} - {\text{EMS}}_{0} }}{{{\text{EMS}}_{0} }} \\ &\quad= \frac{{\left( {p_{0} + \Delta p} \right)\left( {d_{0} + \Delta d} \right)\left( {e_{0} + \Delta e} \right)\left( {i_{0} + \Delta i} \right) - p_{0} \cdot d_{0} \cdot e_{0} \cdot i_{0} }}{{{\text{EMS}}_{0} }} \end{aligned}$$

and extended as

3 $$\begin{aligned} \Delta {\text{EMS}}& = \Delta pd_{0} e_{0} i_{0} + p_{0} \Delta de_{0} i_{0} + p_{0} d_{0} \Delta ei_{0} \\ &\quad+ p_{0} d_{0} e_{0} \Delta i + \ldots + \Delta p\Delta de_{0} i_{0} + \ldots \\ &\quad+ \Delta p\Delta d\Delta ei_{0} + \ldots + \Delta p\Delta d\Delta e\Delta i, \end{aligned}$$

where the first four items have only one factor of change; the remaining items demonstrate the interaction of these factors, assuming that the contribution of each factor to the interaction sum is equal. The change in CO_2_ emissions can be then decomposed into the sum of the contribution of four factors, namely the contribution of population factor, $C_{{\text{p}}}$, the per-capita production factor, $C_{{\text{d}}}$, the energy intensity factor, $C_{{\text{e}}}$, and the emission intensity factor,$C_{i}$.

4 $$\Delta {\text{EMS}} = C_{{\text{p}}} + C_{{\text{d}}} + C_{{\text{e}}} + C_{{\text{i}}}$$

By using the factor of energy intensity $e$ as an example, its contribution $C_{{\text{e}}}$ can be formulated as

5 $$\begin{aligned} C_{{\text{e}}} &= p_{0} d_{0} \Delta ei_{0} + \frac{1}{2}\left( {\Delta pd_{0} \Delta ei_{0} + p_{0} \Delta d\Delta ei_{0} + p_{0} d_{0} \Delta e\Delta i} \right) \\ &\quad+ \frac{1}{3}\left( {\Delta p\Delta d\Delta ei_{0} + p_{0} \Delta d\Delta e\Delta i + \Delta pd_{0} \Delta e\Delta i} \right) \\ &\quad+ \frac{1}{4}\Delta p\Delta d\Delta e\Delta i \end{aligned}$$

The unit and main final products of four selected sub-sectors are shown in Table 1.

**Table 1:** Representation of sub-sectors of the industry sector in Japan

Variable	Unit	Sectoral boundary
Production|Cement	Mt/yr	Manufacture of cement, lime, and plaster
Production|Chemicals	Original unit in each model	Manufacture of basic chemicals, chemical products
Production|Pulp and Paper	Mt/yr	Manufacture of paper and paper products, publishing, printing, and reproduction of recorded media
Production|Steel	Mt/yr	Manufacture and casting of basic iron and steel

The non-ferrous metals sector has a wide range of final products which are not reported in a same unit of energy service demand by all model teams, it is removed from sub-sector-level result figures

Considering their either high emission level/intensity or difficulty of further emission abatement, the sub-sector cement, steel, pulp and paper, and chemicals reported by participating model teams are included in this paper.

### Sub-sectoral technologies

The main low-carbon technologies modeled in the selected industry sub-sectors in the participating models are listed in Table 2.

**Table 2:** H_2_, Biomass, CCS, and other low-carbon industry technologies included in participating models

	Steel	Cement	Chemicals	Pulp and Paper
AIM/Hub-Japan	EAF, CCS	CCS	CCS	Biomass for energy
AIM/Enduse-Japan	EAF, CCS	CCS		Biomass for energy
IEEJ_Japan 2017	EAF, Hydrogen reduction, CCS	CCS	H_2_	Biomass for energy
TIMES-Japan	EAF, Hydrogen reduction, CCS	CCS	H_2_ meeting generic high-temperature heat demands mixed with natural gas, High temperature heat pump	Biomass for energy

Electric Arc Furnace (EAF); H_2_ in IEEJ_Japan 2017 is based on imports; H2 in TIME-Japan can come from both domestic production and imports

## Results

### Final energy of the industry sector in Japan

**Fig. 2:**
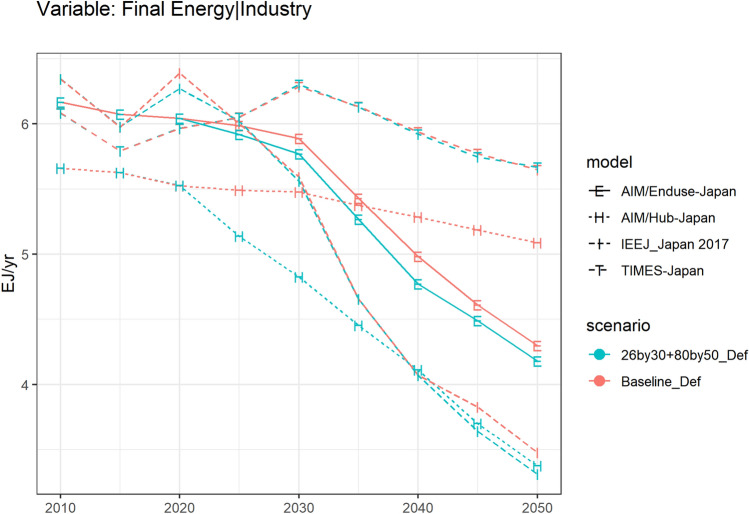
Final energy of the industry sector in Japan since 2010

Figure 2 shows the key variable, final energy of the industry sector under two main scenarios, baseline (Baseline_Def) and NDC&MCS scenario (26% emissions reduction by 2030 and 80% emissions reduction by 2050, 26by30 + 80by50_Def).

Models show different industry shares even for the base year. This variation is partially explained by the difference in the industrial energy coverage, emission coverage, and their databases used. Models use both the energy balance of the International Energy Agency the comprehensive energy statistics compiled by METI. In fact, these databases disagree on the industry share of final energy (see Table ESM i in this paper, and Sugiyama et al. (2021) for more on this point).

Figure 2 shows the final energy of the industry sector from 2010 to 2050. Under the 80% reduction constraint, the long-term energy consumption varies among models, even among all PE models. A 47.8% decline in 2050 can be observed in IEEJ_Japan 2017 compared to the 2010 level, similarly a 32.2% decline in AIM/Enduse-Japan, and a 6.9% decline in TIMES-Japan. Such variation can be caused by the variation of these PE models in base years, in the treatment of external drivers, the coverage of industrial energy, and thus vary in the mitigation measures preferences in the 26by30 + 80by50_Def scenario results. However, all PE models show a similar small gap between the Baseline_Def and 26by30 + 80by50_Def. Extra cut down of energy consumption to achieve the NDC&MCS goal can be expected as limited.

**Fig. 3:**
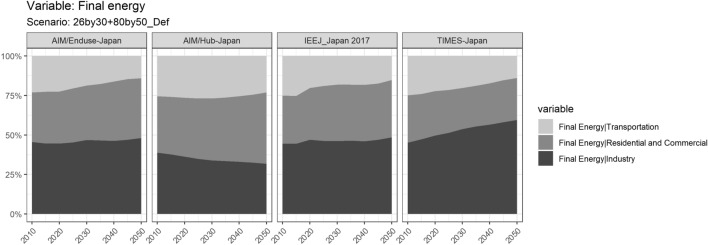
Long term changes of sectoral final energy in Japan under NDC&MDS scenario

The share of industry in Japan’s national final energy is shown in Fig. 3. According to all PE models, around half of the total final energy consumption will be contributed by the industry sector if NDC&MCS goal is achieved, which is a high number given the context that G7 average in 2016 was 19.7% and OECD 21.7% (IEA, 2016). Moreover, the share of the industry sector increases by 2050 in all PE models. In the GE model, this share decreases as the total final energy consumption does not reduce as much as other PE models.

**Fig. 4:**
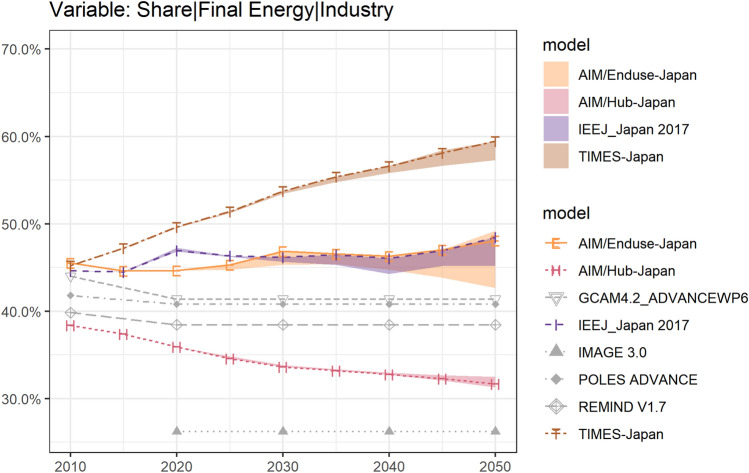
Ranges of industry’s share in final energy under selected scenarios: results from Japan and global models. Source: Regarding the results from EMF35 JMIP model teams, ribbons show the ranges under main scenarios (Baseline_Def, 26by30 + 80by50_Def, 26by30 + 80by50_NoCCS) and lines show the value under 26by30 + 80by50_Def. Regarding the results from global models, source from ADVANCE Synthesis Scenario Database (version 1.0), scenario 2030_Med2C (limit cumulative 2011–2100 CO2 emissions to 1600 GtCO2; more likely than not to stay below 2 °C; implementing without strengthening until 2030), project conducted during 2013–2016. Industry’s share in final energy under more scenarios see Fig. ESM iii

To place Japan’s industry in a broader context, Fig. 4 presents the data from global models in the ADVANCE project (Advanced Model Development and Validation for the Improved Analysis of Costs and Impacts of Mitigation Policies) in addition to the EMF 35 JMIP results. The gray lines show the results of the industry’s share in final energy from global model teams. Although the ADVANCE Synthesis Scenario Database (version 1.0) was conducted earlier during 2013–2016, also the scenario 2030_Med2C is not perfectly comparable with EMF 35 JMIP scenarios, the results are still good references, as these models consider the position of Japan in the global economy, where less attention is paid in EMF 35 JMIP participating models. Based on the global emission restrictions, global models give a lower estimation of the final energy industry share. They reported the emission reduction rate in Japan’s industry sector in 2050 with a range of 35.6% (GCAM4.2_ADVANCEWP6) to 58.3% (IMAGE 3.0, see Fig. ESM iii) compared to the 2010 level, also less than the expectation of model teams from Japan (50.0% to 69.4% reduction). Given such conditions, the results from 3 of all 4 JMIP models show that the industry’s share in Japan will stay still after 2020 and reach around 40 percent by 2050 and, still higher than the estimation of the OECD average from IPCC AR5 (Sugiyama et al. 2019). Compared to these reference models from institutions other than Japan, JMIP PE models show higher results in the final energy industry share (among which the highest 59.5% under 26by30 + 80by50_Def, from TIMES-Japan), closer to the world average rather than OECD countries.

### CO_2_ emissions generated from the industry sector in Japan

**Fig. 5:**
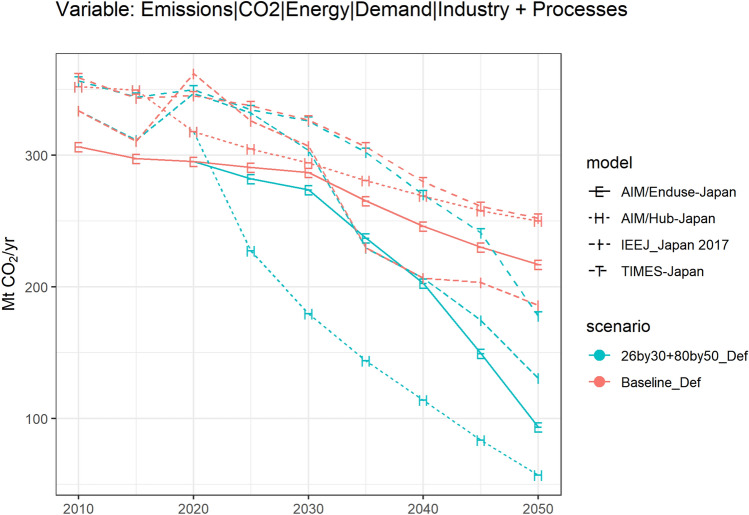
CO_2_ emissions of the industry sector in Japan since 2010

The corresponding CO_2_ emissions of the industry sector under the baseline and the 26by30 + 80by50_Def scenarios are shown in Fig. 5. The variable shows the sum emissions generated from the energy use in the industry sector and from industrial processes.

Compared to the final energy of the industry sector in Fig. 2, the variations in CO_2_ emissions among models are smaller. Under the 26by30 + 80by50_Def scenario, an 83.4% emission reduction in the industry sector in 2050 can be observed in AIM/Hub-Japan compared to the 2010 level, similarly a 69.4% decline in AIM/Enduse-Japan, a 60.8% decline in IEEJ_Japan 2017, and a 50.0% decline in TIMES-Japan. To reach an 80% emission reduction goal in total for all sectors, model teams have different expectations of the emissions reduction efforts of industries.

**Fig. 6:**
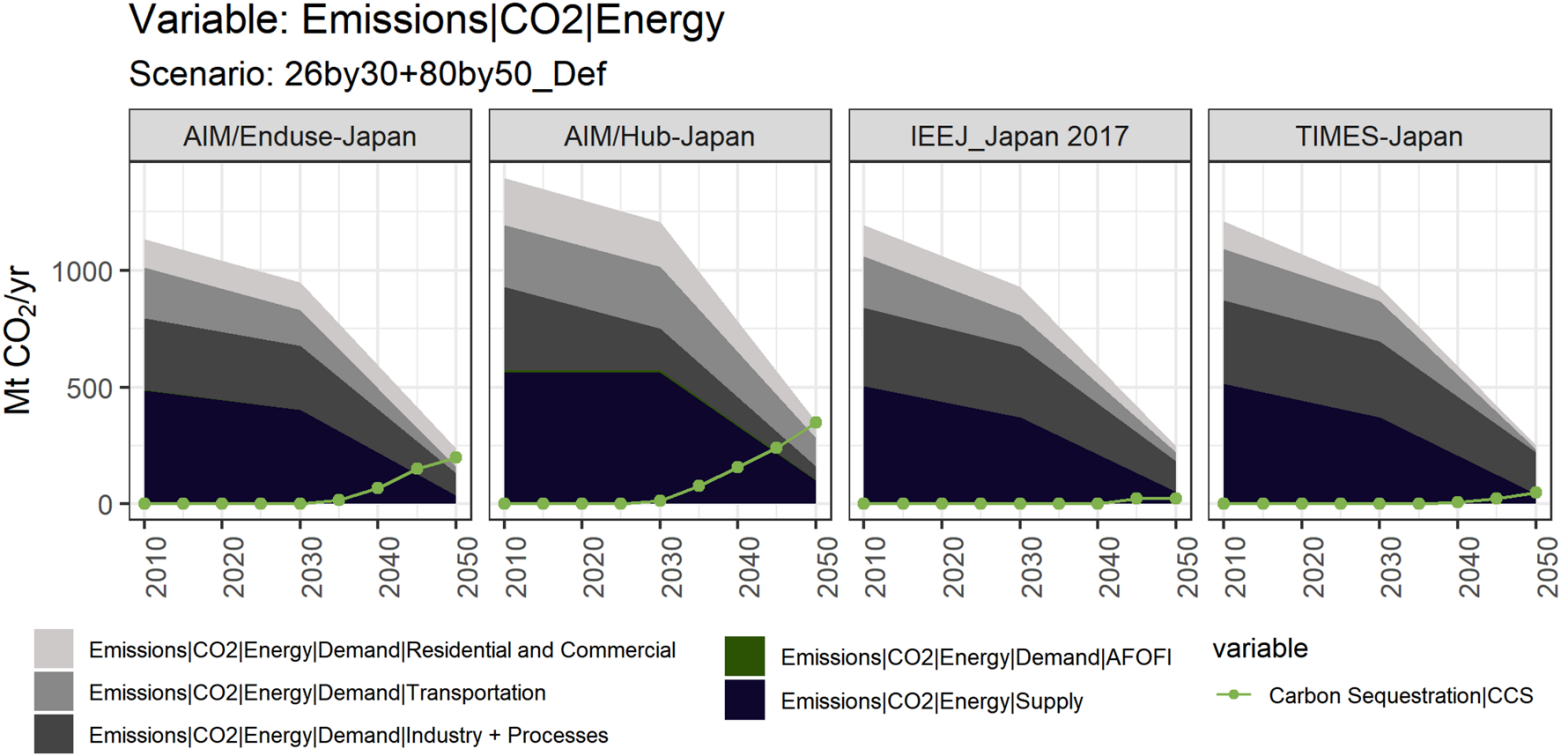
Long term changes of sectoral CO_2_ emissions in Japan and the contribution of carbon sequestration under NDC&MCS scenario

Similar to the structure of sectoral final energy, the industry sector occupies the largest share of demand-side total emissions in all PE models (See Fig. 6). The implementation of CCS in industry largely varies among models. The 80% emission reduction by 2050 will be contributed significantly by CCS, especially the CCS of fossil fuels according to the results from AIM/Hub-Japan and AIM/Enduse-Japan. On the other hand, more implementation of CCS does not seem very necessary to achieve the NDC-MCS goal according to the results from IEEJ_Japan 2017 and TIMES-Japan, among which a certain share of emissions generated from industry-related activities would be captured in TIMES-Japan.

Regarding which sector would cut down more emissions, in half of the participating models (AIM/Enduse-Japan and TIMES-Japan), the transportation sector shows a larger potential in the emission reduction with lower marginal costs, and its absolute number of reduction exceeds the industry sector. In the other half of the models (AIM/Hub-Japan and IEEJ_Japan 2017), a larger burden of emission mitigation will go to the industry sector, shown as a reduction in industry’s annual emissions overweighs others. No matter to which sector such priority of emission reduction burden would go, the results of JMIP suggest that annual CO_2_ emission in the industry sector should at least cut around 150 Mt in 2050 compared to the 2010 level. How such a cut will be achieved, namely to what extent fuel switching in industries works, which sub-sectors should decarbonize more, or other mitigation measures that have not been decently modeled in this project, would be investigated in the next sections. Among all participating models, the GE model shows the largest net emission reduction in the industry sectors.

### Decomposition by source

**Fig. 7:**
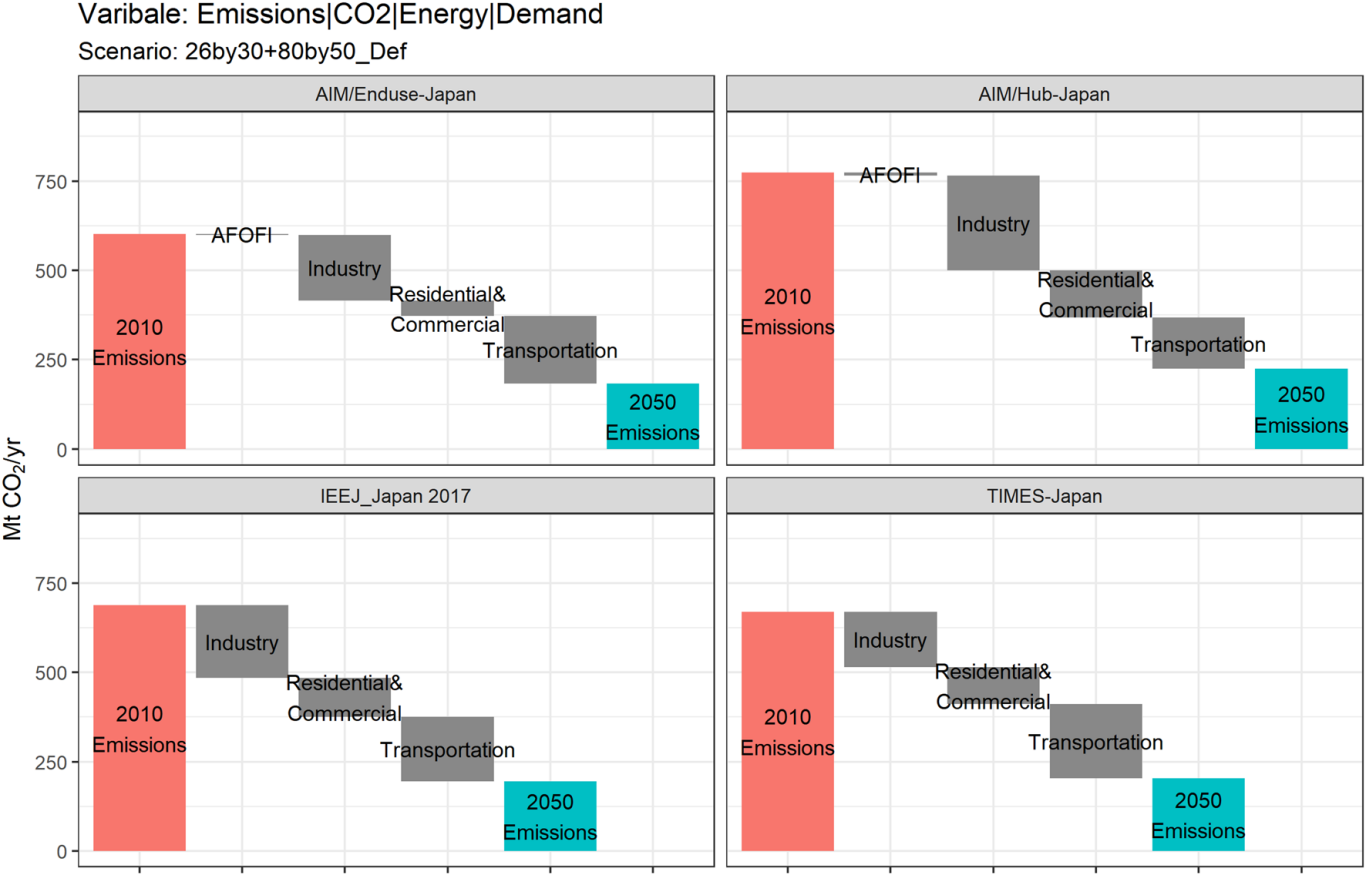
Industry’s contribution to the reduction in annual demand-side emissions under NDC&MCS scenario

The decomposition of the industry’s final energy by source is shown in Fig. 7, compared with the same decomposition in other sectors.

According to PE models, the industry sector may still rely on the energy consumption of solids in 2050, which is a relatively larger share compared to other sectors. Among such decomposition of consumption, only around 10% will be biomass, and the rest still coal. Moreover, all PE models report very similar results of the industrial electrification level. Under the NDC&MCS scenario, the share of electricity consumption in the industry sector will remain at a relatively low level and slightly increase during 2010–2050. Factors determining such an electrification rate are numerous and would need to be analyzed separately in each sector (Sakamoto et al. 2021). The modeling of electricity technologies in industries (e.g., electric arc furnaces in steelmaking, or more generally the use of electricity to meet industrial heat demands), as well as the price and changes in prices of such electricity technologies, may affect the result of electrification rate. Furthermore, the large-scale introduction of electricity-based facilities may sharply increase the industrial electricity consumption and exert more pressure on the electricity supply. However, the manner in which energy service demands react to such changes with respect to the availability of energy supply cannot be solved simultaneously in PE models with exogenous energy service demands. On the other hand, the switch from fossil fuels to hydrogen in industries is less costly in terms of system mortification, such as fewer changes in sensors, controls, and labor skills (ICEF, 2019). However, its introduction in the industry sector will be limited according to Fig. 7.

Overall, according to the results from PE models, the rise in the electrification rate and the introduction of biomass use in industries by 2050 will still be limited, suggesting a low possibility of large-scale fuel switching or end-use technology substitution in production processes in Japan. How industries can benefit from an increasingly low-carbon energy supply remains a pressing issue.

### Decomposition by sub-sector

**Fig. 8:**
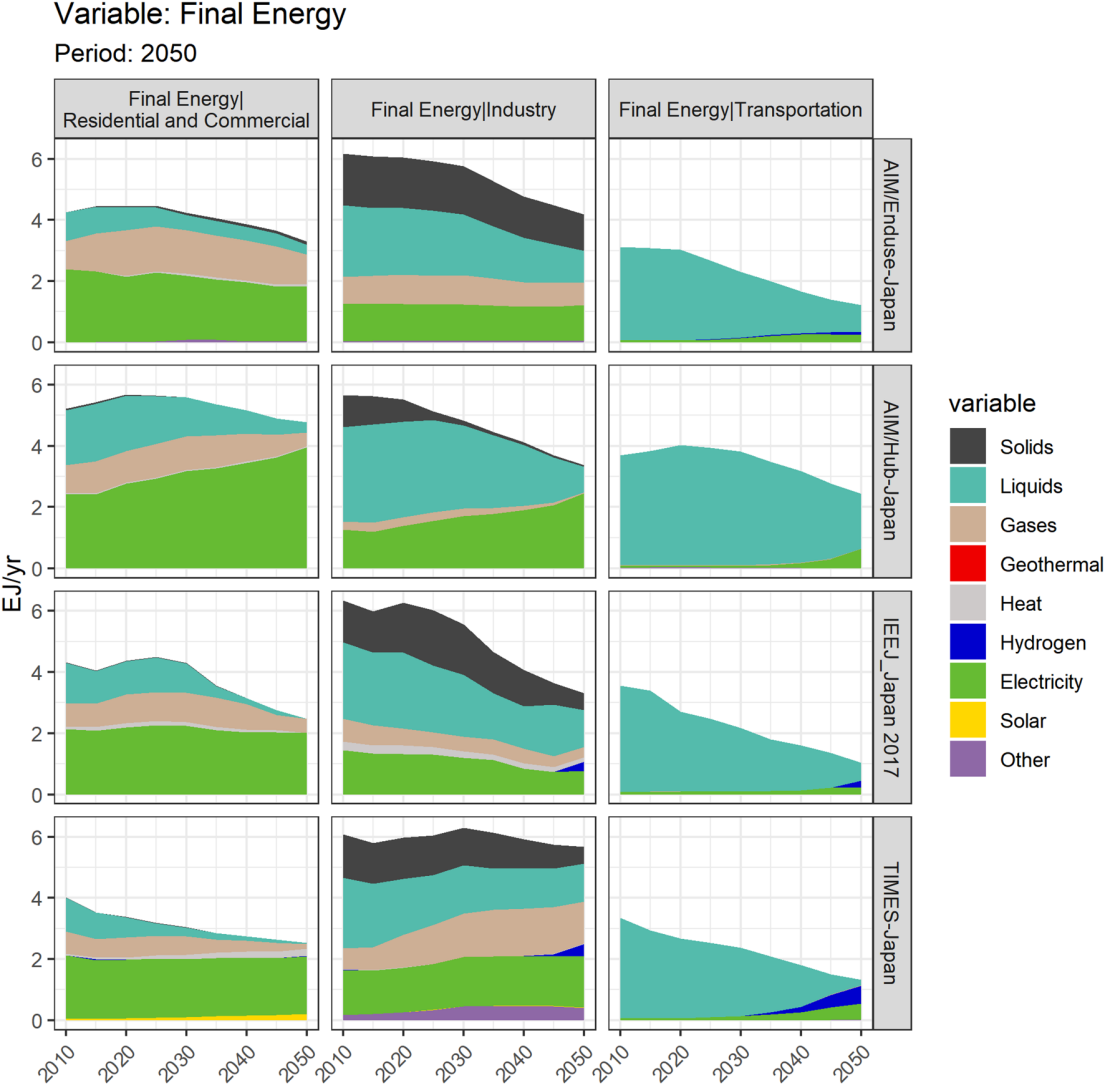
Sectoral final energy by source under NDC&MCS scenario. Notes: the decomposition by source under more scenarios in 2050 see Fig. ESM iv

Cement, chemicals, pulp and paper, steel, the final energy and CO_2_ emissions of the four selected industry sub-sectors are shown in Fig. 8.

**Fig. 9:**
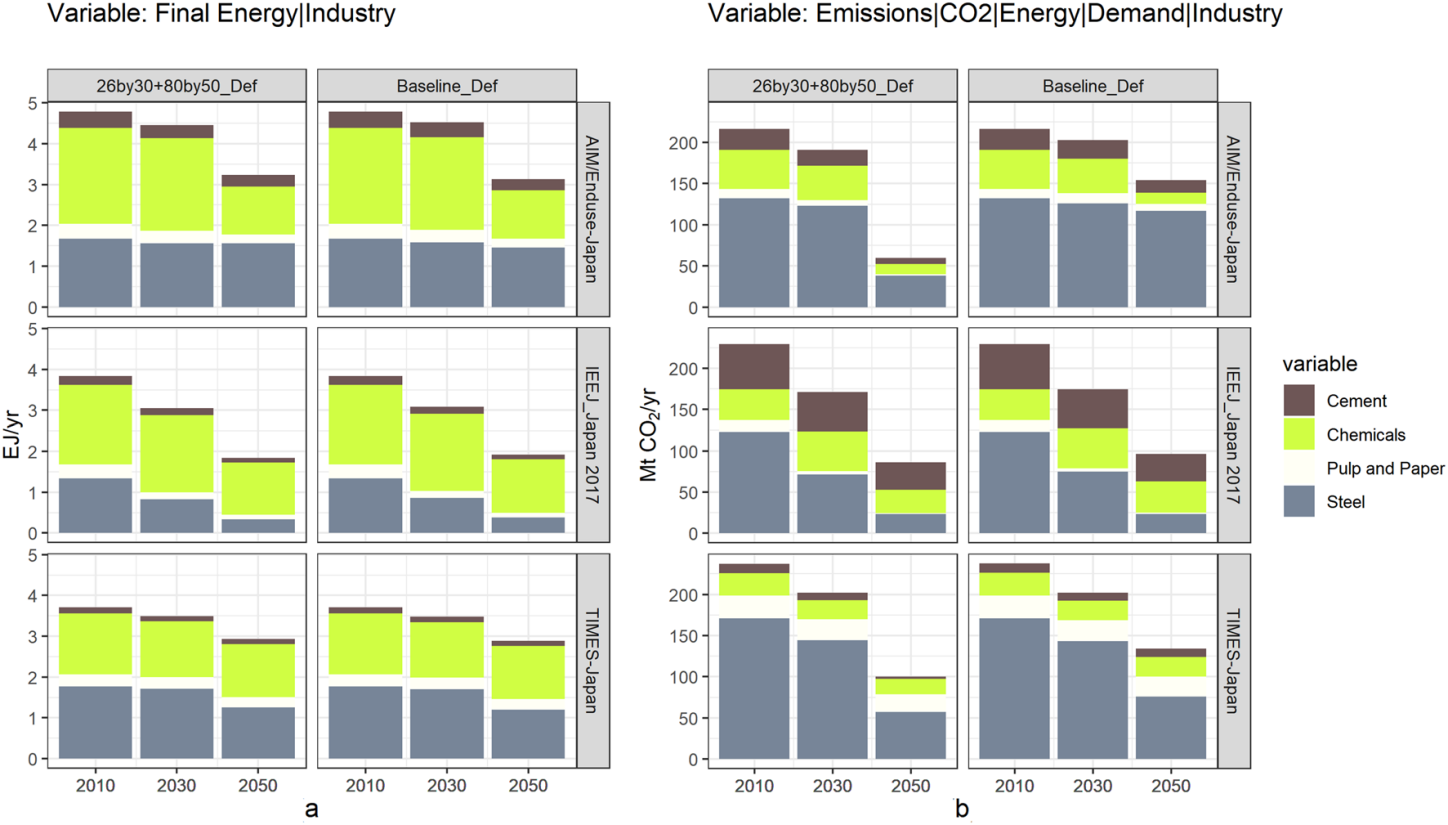
Sub-sectoral industry’s final energy (**a**) and CO_2_ emissions (**b**) by sub-sector under NDC&MCS scenario. Notes: the decomposition by subsector under more scenarios in 2050 see Fig. ESM vi; emissions in **b** only track emissions from energy sources and do not include emissions generated from industrial processes (except the steel sub-sector in TIMES-Japan)

The gap of sub-sectoral final energy between NDC&MCS and baseline scenarios is small in all sub-sectors except steel, so is the gap of sub-sectoral CO_2_ emissions. The potentials of both emission reduction and energy conservation of these sub-sectors would be limited. On the other hand, sub-sectoral final energy and CO_2_ emissions do not share a similar structure. In the cement sub-sector, the high emission intensity and the large number of emissions would be generated from production processes, shown as a small share in final energy and a larger share in CO_2_ emissions. As mentioned in Fig. 9, annual CO_2_ emission in the industry sector should at least cut around 150 Mt in 2050, among which around 100 Mt cut would be the mission of the steel sub-sector. A large share in final energy and a larger share in CO_2_ emissions, together with such a large gap between emission levels in 2050 and 2010, again emphasized the key position of steelmaking decarbonization to the achievement of Japan’s NDC&MDS goal.

The sub-sectoral CO_2_ emissions under more scenarios also see Fig. ESM ii. The selected scenarios can examine the impacts of two mitigation measures in the industry sector, CCS and lower energy service demands. Both final energy and CO_2_ emissions are reported as the lowest value under LoDemInd scenarios among all scenarios in nearly all sub-sectors and models. In the steel sub-sector, around 50–60 Mt emissions will be reduced (compared to the baseline scenario) by halving steelmaking’s energy service demand.

The other mitigation measure, CCS, is modeled in the steel and cement sub-sectors in all participating models. In AIM/Enduse-Japan, the emission of steelmaking would be much higher under the 26by30 + 80by50_NoCCS scenario than under the 26by30 + 80by50_Def scenario, especially after 2030. Such a difference indicates the importance of CCS to the decarbonization of steelmaking in AIM/Enduse-Japan. In TIMES-Japan and IEEJ_Japan 2017, the emission of steelmaking under the 26by30 + 80by50_NoCCS scenario would be lower or nearly the same under the 26by30 + 80by50_Def scenario, indicating the limited contribution of CCS in steelmaking decarbonization in these two models. The emission reduction would be achieved by the introduction of hydrogen technologies in steelmaking in TIMES-Japan (after 2040, shown in Fig. 7). While in the cement sub-sector, a larger impact of CCS can be observed in TIMES-Japan.

**Fig. 10:**
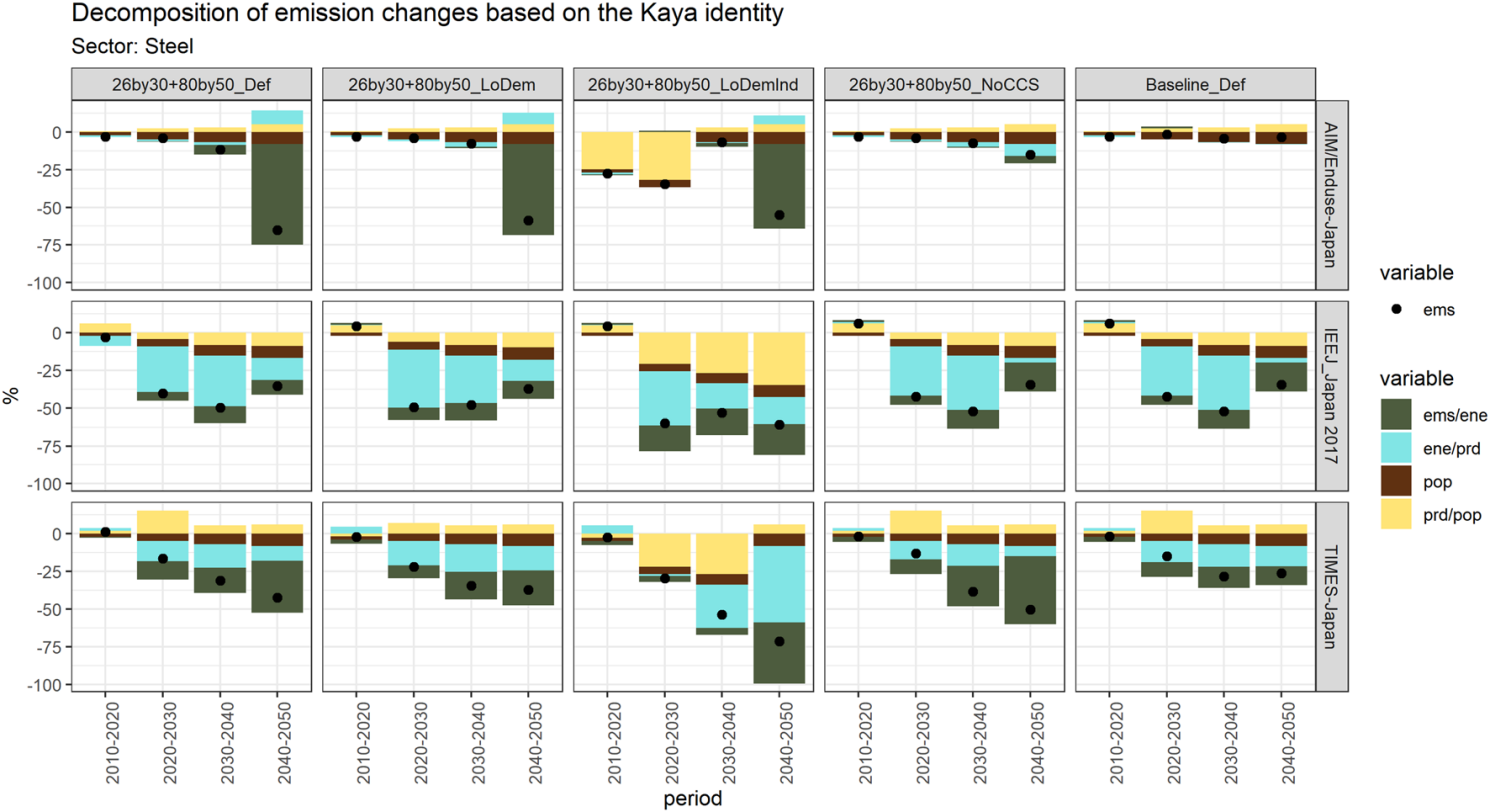
Decomposition of emission changes based on the Kaya identity: the steel sub-sector. Notes: the decomposition results of all sub-sectors see ESM v

Considering the key role of steelmaking, a decomposition considering more scenarios is conducted in this sub-sector, shown in Fig. 10. The decomposition reveals how much each factor, namely changes in final demands for industrial products, energy efficiency improvement, and emission intensity reduction, would contribute to the changes of sub-sectoral emission (results of all sub-sectors see Fig. ESM v). According to the results, the contribution of emission intensity (green bar) will overweigh the contribution of energy efficiency (blue bar) after 2030, especially in the steel and cement sub-sector.

From the temporal perspective, two of the three models report that significant emission reductions in the steel sector may occur from 2040 to 2050, instead of a continuous reduction after 2020. From the perspective of factors, the impact of emission intensity factor would concentrate in the period 2040–2050, while the energy efficiency factor would keep functioning from 2020, which is along with the decomposition result of all sub-sectors.

Regarding the contribution of energy efficiency improvement, its effect on emission reduction is significant during 2020–2040 in IEEJ_Japan 2017, while it is smaller, but still exists, in TIMES-Japan during the whole period. In AIM/Enduse-Japan, the contribution of would be lower in the period 2040–2050 due to the introduction of more CCS. Models hold different views but all agree that even if there is no CCS implemented, the steelmaking decarbonization cannot count on energy saving after 2040. Regarding the contribution of emission intensity reduction, it is reported in IEEJ_Japan 2017 that certain contributions would exist throughout the whole period, in TIMES-Japan mainly after 2030, and in AIM/Enduse-Japan huge contributions only concentrated in the period 2040–2050. As mentioned, this is a reflection of the CCS implementation in AIM/Enduse-Japan and the more introduction of hydrogen technologies after 2040 in TIMES-Japan.

Regarding the contribution of final product demand changes from 2010 to 2016, the decrease in production volume has not been as significant as other industrial materials such as non-ferrous metals and cement (Oda and Akimoto 2019).The long-term expectation of the production of steel also considers global assumptions (Nameki and Moriguchi 2014) that may affect total domestic production, as well as the potential of recyclable scraps (Kawase and Matsuoka 2015) that may affect the introduction of EAF capacity. The estimation of AIM/Enduse-Japan and TIMES-Japan shows that steel production may slightly but steadily increase, while this growth may cease in 2020, drop steadily afterward, and lead to the reduction in emissions in IEEJ_Japan 2017. All models report the largest emission mitigation led by a reduction in production under the low industry demand scenario in nearly all periods. Such reduction would ease the pressure of energy conservation, although TIMES-Japan reports that such a decrease in steel demand and the decarbonization by such lower demand would not continue after 2040. Moreover, the marginal abatement cost of CO_2_ emissions would be the lowest under the low industry demand scenario, followed by the building sector and the transportation sector (the results of carbon price see Fig. ESM vii).

## Discussion

Robust assessment of long-term decarbonization of the industry sector relies on consideration of prospect low carbon technologies. This multi-model analysis revealed key limitations and aspects to improve such assessment in terms of the coverage of decarbonization technologies. CCS is applicable in all the participating models, whereas the hydrogen technologies in steelmaking are only included in some models. The use of biomass as fuel or feedstock in the chemical sub-sector is not included in all the participating models. These limitations are barriers to the representation of fuel switching in the industry sector. Moreover, the performance of mitigation measures other than energy conservation in the future industries cannot be revealed if the currently unmatured technologies have not been modeled in the first place. Widening the range of end-use technologies and devices may greatly improve the modeling of IAMs. Table 3 shows the steelmaking and cement technologies worldwide, in the EU, in Japan, and in all participating models.

**Table 3:** Modeling of industry technologies in IAMs: in steel and in cement sub-sector

Technologies worldwide (steel sub-sector)	GHG reduction potential	TRL	Technologies (under development included) in EU	Technologies (under development included) in Japan	Technologies covered by JMIP participating models
Current EAF (depends on electricity intensity)	Up to 99%				All
BF-BOF w/ top gas recirculation & CCUS (Leeson et al. 2017; Axelson et al. 2018; Birat 2011)	60%	High	Advanced direct reduction with CCS; EC 2013)	CCUS (recovery/recycling from byproduct gases; JISF 2019)	All
HIsarna with concentrated CCUS (Axelson et al. 2018)	80–90%	Medium	HIsarna (CCS; EECRsteel 2011)	Ferro-coke process (NEDO 2019)	
Hydrogen direct reduced iron (Fischedick et al., 2014; Vogl et al., 2018)	99%	Medium	Hydrogen direct reduced iron	Internal hydrogen (JISF 2019)	IEEJ_Japan 2017, TIMES-Japan
Aqueous and Molten Oxide electrolysis (Axelson et al., 2018; Fischedick et al., 2014)	99%	Low	Electrolysis process (EC 2013)		

Technologies worldwide (cement sub-sector)	GHG reduction potential	TRL	Technologies (under development included) in EU	Technologies (under development included) in Japan	Technologies covered by JMIP participating models
Clinker substitution (e.g., limestone + calcined clays)	40–50%	High	Further reduction of clinker content (ECRA 2017)	Improve efficiency (cooler, kiln, preheater, etc.; JCA 2013)	All
Alternative lower GHG fuels (e.g., waste biofuels and hydrogen)	40%	High	Alternative fuels, Fuel switching, waste heat recovery (steam, ORC, Kalina Cycle; ECRA 2017)	Alternative fuels and waste heat recovery (JCA 2013)	All
CCUS for process heating & Calcination-carbonation cycle (Moore 2017; Leeson et al. 2017)	99% calc., < = 90% heat	Medium	Post-combustion capture	Calcination-carbonation cycle	All
Electrification of the calciner (Hills et al. 2016, 2017)	60%	Medium			
Magnesium or ultramafic cements (Lehne and Preston 2018; Scrivener et al. 2018; Gartner and Sui, 2017)	Can be negative	Low			

Notes: Technology readiness level (TRL). For the steel sub-sector, the current global average emission intensity is 1.83 tCO_2_-eq/t (Worldsteel 2019); the details of more conventional mitigation measures (e.g., coke dry quechning, top pressure recovery turbine, etc.) are not included in this table. For the cement sub-sector, the current global average emission intensity is 0.55 tCO_2_-eq/t (ECRA 2017); the details of more conventional mitigation measures (e.g., pre-heaters) are not included in this table.

Although the technology development in industries has been included in some reports in Japan (NEDO 2018; METI 2019c), the range of the categories of current technologies/practices in Japan is smaller than that of the EU, and even smaller when represented in JMIP IAMs. According to the mitigation scenarios for the steel industry conducted by the Japan Iron and Steel Federation, hydrogen-reduction, CCS, and CCU are included in the most optimistic scenario by the steel industry (JISF 2019). However, technologies with low technology readiness levels but high reduction potential, such as aqueous (e.g., developed during the project SIDERWIN) and molten oxide electrolysis (e.g., developed by Boston Metals) in steelmaking, electrification of the calciner (e.g., developed during the project LEILAC), and magnesium or ultramafic cement in cement-making has not attracted large-scale interests of either participating models or industry stakeholders in Japan. The modeling of such sub-sectors in a wider range of periods, such as by 2100, is also worth considering.

Also, the uptake of hydrogen, specifically the direct reduced iron technology has not been included in all model teams. Recently, three main producer companies in Japan and two in Europe have raised their total investment in coal-free steelmaking technologies, reaching 264.7 billion yen in 2019, an 14% increase compared to the 2015 level (Nikkei 2020). In our future works, another set of NoHydrogen scenarios will help investigate the contribution of hydrogen introduction in energy demand sectors.

So far, over 3/4^th^ of the CO_2_ capture capacity that has been built in the past decade and that is currently operational worldwide is in low-cost processes (such as hydrogen production-related processes, gas processing, etc.) instead of industries, wherein the capture and use of CO_2_ would be economically and technically challenging (IEA 2019). In Japan’s industries, CCS has been regarded as a technocratic approach that fully relies on consensus among political elites and experts (Asayama and Ishii 2014). To bridge the gap in the current status and future deployment of CCS capacity, it is essential to stimulate early investments in steel and cement sub-sectors. Such investment can be supported by targeted policy instruments such as tax credits or market-based schemes. Before these steps are taken, there should be public awareness of carbon capture’s necessity and the establishment of a grand design that focuses on the type of CCUS technologies that should be introduced in specific sectors (as discussed in an early stage in the Study Group for Innovative Environmental Innovation Strategy, METI 2019c), both of which require efforts from the modeling communities.

Figure 4 compares the Japanese and global IAM results with the industry’s share in the final energy consumption. Although an analysis of the reasons that lead to such differences between the Japanese and global IAM results was conducted in this paper, such differences re-emphasize the necessity of model intercomparison projects (van Sluisveld et al. 2019). Policymakers will have a more holistic view of the models have better access to parameters of local activities and a better understanding of global networks. Regarding the sensitivity of scenario parameters, the ranges of the key variables (i.e., industrial final energy) under demand scenarios and policy scenarios are shown in Fig. ESM vii.

## Conclusion

In this paper, the data from four energy economic and integrated assessment models were utilized to explore climate mitigation scenarios of Japan’s industry by 2050, including its final energy and CO_2_ emissions, their long term changes and structures, as well as the impacts of several industrial mitigation measures. This was followed by a decomposition of emission changes based on the Kaya identity to investigate what how Japan’s industrial decarbonization would be driven. The results show that:

The industry sector dominates Japan’s total final energy consumption. By 2050, its share will increase in all the partial equilibrium models, further indicating the difficulty in achieving industrial decarbonization by improving energy efficiencies. The general equilibrium model shows the largest net emission reduction in the industry sector. These results of JMIP suggest that, in order to achieve the Nationally Determined Contribution and Mid-Century Strategy goal of Japan, a large cut in the annual CO_2_ emission in the industry sector would be inevitable. Compared to other sectors, the industry sector may still rely on solids in 2050, as raw materials in production as well as fuels to meet the industrial heat demand. Under the mitigation scenarios, the rise in the electrification rate and the introduction of biomass use in industries will still be limited (electrification rate up to around 30% in all PE models), suggesting a low possibility of large-scale fuel switching or end-use technology revolution in production processes in Japan.

Regarding the mitigation measure energy saving, the contribution of emission intensity reduction to the industrial decarbonization will overweigh the contribution of energy efficiency improvement after 2030, especially in the steel sub-sector and cement sub-sector. Decarbonization in steelmaking would be key to the achievement of Japan’s national emission reduction goal. Such a cut in steelmaking can be achieved by the implementation of CCS or more introduction of hydrogen technologies after 2040. Decarbonization in steelmaking cannot count on extra energy conservation after 2040. Low demand for energy services in the industry sector may largely decrease the marginal abatement cost of CO_2_ emissions (Sugiyama et al. 2021).

Stocktaking of the current modeling practice helps us see the limitation of how the current modeling investigates the crucial concerns of industries in Japan. A wider range of end-use technologies and devices, including technologies with low technology readiness levels, can be considered as alternative options in energy-related integrated assessment models that are highly encouraged. The discussion in this paper also leads to more research questions, such as how industries can benefit from increasingly low-carbon energy supply, or to support such a transition in energy supply, how would the demand for industrial products and services change.

## Supplementary Information

Below is the link to the electronic supplementary material.

Supplementary file1 (DOCX 1102 KB)

## References

[CR1] Åhman M, Nilsson LJ, Johansson B (2017) Global climate policy and deep decarbonization of energy-intensive industries energy-intensive industries, 3062. Doi: 10.1080/14693062.2016.1167009

[CR2] Akashi O, Hijioka Y, Masui T, Hanaoka T, Kainuma M (2012). GHG emission scenarios in Asia and the world: the key technologies for significant reduction. Energy Econ.

[CR3] Ang BW (2000). A survey of index decomposition analysis in energy and environmental studies. Energy.

[CR4] Asayama S, Ishii A (2014). Media representations and governance of CCS: framings and policy implications of Japanese newspaper's coverage. Soc Tech Res Essay Collect.

[CR5] Ashina S, Fujino J, Masui T, Ehara T, Hibino G (2012). A roadmap towards a low-carbon society in Japan using backcasting methodology: Feasible pathways for achieving an 80% reduction in CO_2_ emissions by 2050. Energy Policy.

[CR6] Axelson M, Robson I, Khandekar G, Wyns T (2018) Breaking through: Industrial Low-CO_2_ Technologies on the Horizon. 92. www.ies.be/Breaking-Through_Report_13072018

[CR7] Bataille C, Åhman M, Neuhoff K, Nilsson LJ, Fischedick M, Lechtenböhmer S (2018). A review of technology and policy deep decarbonization pathway options for making energy-intensive industry production consistent with the Paris Agreement. J Clean Prod.

[CR8] Birat J (2011). The sustainability footprint of steelmaking. Steel Times Int.

[CR9] Davis SJ, Lewis NS, Shaner M, Aggarwal S, Arent D, Azevedo IL (2018). Net-zero emissions energy systems. Science.

[CR10] ECRA (European Cement Research Academy, 2017). CSI/ECRA-technology papers 2017: development of state of the art techniques in cement manufacturing: trying to look ahead. http://docs.wbcsd.org/2017/06/CSI_ECRA_Technology_Papers_2017.pdf

[CR11] EECRstee. (2011) Proceedings of the first international conference on energy efficiency and CO_2_ reduction in the steel industry, HIsarna Pilot Plant Project, Meijer, K., Guenther, C., Dry, R.J., 27 June to 1 July 2011, Dusseldorf, 2011.

[CR12] Ehrlich PR, Holdren JP (1971). Impact of population growth. Obstet Gynecol Surv.

[CR13] European Commission (2013) Technology map of the european strategic energy technology plan (SET-Plan). https://ec.europa.eu/jrc/en/printpdf/155402

[CR14] Fischedick M, Marzinkowski J, Winzer P, Weigel M (2014). Techno-economic evaluation of innovative steel production technologies. J Clean Prod.

[CR15] Fujimori S, Kainuma M, Masui T, Hasegawa T, Dai H (2014). The effectiveness of energy service demand reduction: a scenario analysis of global climate change mitigation. Energy Policy.

[CR16] Gartner E, Sui T (2017). Alternative cement clinkers. Cem Concr Res.

[CR17] GoJ (Government of Japan, 2015). Submission of Japan's intended nationally determined contribution (INDC). http://www4.unfccc.int/submissions/INDC/ Published%20Documents/Japan/1/20150717_Japan's%20INDC.pdf.

[CR18] GoJ (2016) The plan for global warming countermeasure. http://www.env.go.jp/press/files/jp/102816.pdf [in Japanese]

[CR19] Grubler A, Wilson C, Bento N, Boza-kiss B, Krey V, Mccollum DL (2018). A low energy demand scenario for meeting the 15 °C target and sustainable development goals without negative emission technologies. Nat Energy.

[CR20] Hertwich EG, Coauthors. (2019). Material efficiency strategies to reducing greenhouse gas emissions associated with buildings, vehicles, and electronics—a review. Environ Res Lett.

[CR21] Hills TP, Sceats M, Rennie D, Fennell P (2017). LEILAC: Low cost CO_2_ capture for the cement and lime industries. Energy procedia.

[CR22] Hills T, Leeson D, Florin N, Fennell P (2016). Carbon capture in the cement industry: technologies, progress, and retrofitting. Environ Sci Technol.

[CR23] ICAP (International Carbon Action Partnership) (2020a) Japan—saitama target setting emissions trading system. https://icapcarbonaction.com/en/?option=com_etsmap&task=export&format=pdf&layout=list&systems[]=84

[CR24] ICAP (2020b) Japan—Tokyo cap-and-trade program. https://icapcarbonaction.com/en/?option=com_etsmap&task=export&format=pdf&layout=list&systems[]=51

[CR25] ICEF (Innovation for Cool Earth Forum) (2016) ICEF innovation roadmap 1.0: CO_2_ utilization and ZEB/ZEH roadmaps released at COP22. https://www.icef-forum.org/platform/article_detail.php?article__id=109

[CR26] IEA (International Energy Agency) (2016). World energy balances 2016.

[CR27] IEA (2017a) Energy technology perspectives 2017—executive summary. International Energy Agency (IEA) Publications, 371. Doi: 10.1787/energy_tech-2014-en

[CR28] IEA (2017). Renewable Energy for Industry: From green energy to green materials and fuels.

[CR29] IEA (2019) Tracking industry, IEA, Paris https://www.iea.org/reports/tracking-industry

[CR30] IPCC (Intergovernmental Panel on Climate Change) (2014) Fifth assessment report of the intergovernmental panel on climate change. Cambridge University Press 2014. https://www.ipcc.ch/site/assets/uploads/2018/02/ipcc_wg3_ar5_full.pdf

[CR31] IPCC (2018) Proposed outline of the special report in 2018 on the impacts of global warming of 1.5°C above pre-industrial levels and related global greenhouse gas emission pathways, in the context of strengthening the global response to the threat of climate cha. IPCC - Sr15, 2(October), 17–20. www.environmentalgraphiti.org

[CR32] IPSS (2017) Population Projections for Japan (2017): 2016 to 2065. National Institute of Population and Social Security Research

[CR33] JBF (Japan Business Federation) (2019). KEIDANREN's commitment to a low carbon society fiscal 2018 follow-up results. https://www.keidanren.or.jp/policy/vape.html

[CR34] JBF (2014) Low carbon society action plan independent evaluation committee evaluation report. https://www.keidanren.or.jp/policy/2015/039.pdf

[CR35] JCA (Japan Cement Association) (2013) Cement industry's efforts to combat global warming. http://www.jcassoc.or.jp/seisankankyo/seisan02/pdf/seisan02_01.pdf

[CR36] JCA (2014) Low carbon society action plan phase II (cement industry). http://www.jcassoc.or.jp/cement/4pdf/jg1k_03.pdf

[CR37] JISF (Japan Iron and Steel Federation) (2014) Low carbon society action plan phase II (iron and steel industry). https://www.jisf.or.jp/business/ondanka/kouken/keikaku/documents/141112.pdf

[CR38] JISF (2019) Long-term vision for climate change mitigation A challenge towards zero-carbon steel, 2019. http://www.jisf.or.jp/business/ondanka/zerocarbonsteel/

[CR39] Kawase R, Matsuoka Y (2015) Steel production estimation in Japan with considering global market, Proceedings of the Civil Engineering Society (Environment), vol 71, No. 5, I_383–394. https://www.jstage.jst.go.jp/article/jscejer/71/5/71_I_383/_pdf

[CR40] Kaya Y (1990) Impact of carbon dioxide emission control on GNP growth: interpretation of proposed scenarios. In: paper presented to the IPCC energy and industry subgroup, response strategies working group.

[CR41] Kuramochi T, Ramírez A, Turkenburg W, Faaij A (2012). Comparative assessment of CO_2_ capture technologies for carbon-intensive industrial processes. Prog Energy Combust Sci.

[CR42] Kuramochi T (2016). Assessment of midterm CO_2_ emissions reduction potential in the iron and steel industry : a case of Japan. J Clean Prod.

[CR43] Kuriyama A, Tamura K, Kuramochi T (2019). Can Japan enhance its 2030 greenhouse gas emission reduction targets? Assessment of economic and energy-related assumptions in Japan’s NDC. Energy Policy.

[CR44] LANDES (2011) Product details of CO_2_-SUICOM. https://www.landes.co.jp/product/113

[CR45] Leeson D, Fennell P, Shah N, Petit C, Mac Dowell N (2017). A Techno-economic analysis and systematic review of carbon capture and storage (CCS) applied to the iron and steel, cement, oil refining and pulp and paper industries. Int J Greenh Gas Control.

[CR46] Lehne J, Preston F (2018). Making concrete change innovation in low-carbon cement and concrete. Chatham House Rep.

[CR47] Mccollum DL, Gambhir A, Rogelj J, Wilson C (2020). Energy modellers should explore extremes more. Nat Energy.

[CR48] McMillan C, Boardman R, McKellar M, Sabharwall P, Ruth M, Bragg-Sitton S (2016) Generation and use of thermal energy in the US industrial sector and opportunities to reduce its carbon emissions.

[CR49] METI (Ministry of Economy, Trade, and Industry) (2013) Japan's policy on energy conservation. https://eneken.ieej.or.jp/data/4746.pdf

[CR50] METI (2015a) Top runner program: developing the world's best energy efficient appliance and more. 2015. https://www.enecho.meti.go.jp/category/saving_and_new/saving/data/toprunner2015e.pdf

[CR51] METI (2015b) Chouki enerugi jyukyu mitoshi (long-term energy outlook, in Japanese). The subcommittee on long-term energy supply and demand outlook. https://www.enecho.meti.go.jp/committee/council/basic_policy_subcommittee/mitoshi/pdf/report_01.pdf

[CR52] METI (2016) Long-term global warming counter measure platform (Choki Chikyu Ondanka Taisaku Prattohommu). http://www.meti.go.jp/press/2017/04/20170414006/20170414006-1.pdf.

[CR53] METI (2017) Connected industries Tokyo initiative 2017, https://www.meti.go.jp/policy/mono_info_service/connected_industries/index.html

[CR54] METI (2018) Fifth energy basic plan. https://www.enecho.meti.go.jp/category/others/basic_plan/#head

[CR55] METI (2019a) 2018 annual report on energy (energy white paper), https://www.enecho.meti.go.jp/about/whitepaper/

[CR56] METI (2019b) Large-scale CCS Demonstration Project in Hokkaido Prefecture, Successfully Injects 300,000 Tons of CO2 cumulatively, https://www.meti.go.jp/english/press/2019/1125_004.html

[CR57] METI (2019c) Innovative Environmental Innovation Strategy. https://www.meti.go.jp/shingikai/energy_environment/kankyo_innovation/003.html

[CR58] METI (2020) 2019 Manufacturing white paper (annual report based on article 8 of the basic act on the promotion of basic manufacturing technology). https://www.meti.go.jp/report/whitepaper/mono/2019/index.html

[CR59] MOE (Ministry of the Environment) (2007) Keynote speech by ichiro kamoshita, minister of the environment of Japan. 2007. https://www.env.go.jp/earth/g8/en/meeting/img/Keynote%20Speech%20by%20Ichiro%20Kamoshita%20in%20the%20Session%20on%20Climate%20Change.pdf

[CR60] MOE (2008) Action plan for creating a low-carbon society. http://www.env.go.jp/press/file_view.php?serial=11912&hou_id=10025

[CR61] MOE (2014) Tax to combat global warming. https://www.env.go.jp/policy/tax/faq.html

[CR62] MOE (2016a) Long-term, low-carbon vision. http:// www.env.go.jp/press/103822/105478.pdf

[CR63] MOE (2016b) global warming countermeasures plan (Japanese Cabinet Decision). https://www.env.go.jp/press/102512.html

[CR64] MOE (2019a) Long-term growth strategy based on the Paris Agreement, https://www.env.go.jp/press/106869.html

[CR65] MOE (2019b) 2018 annual report on environment (environment white paper), http://www.env.go.jp/policy/hakusyo/

[CR66] Moore J (2017). Thermal hydrogen: an emissions free hydrocarbon economy. Int J Hydrogen Energy.

[CR67] Nameki M, Moriguchi Y (2014) Recent developments in efforts to reduce GHG emissions in the iron and steel sector and their potential reductions in major producer countries. In: Proceedings of the annual conference of the Japan Institute of Energy, vol 23(0), pp 254–255. Doi: 10.20550/jietaikaiyoushi.23.0_254

[CR68] NEDO (New Energy and Industrial Technology Development Organization) (2018) Open innovation white paper. https://www.nedo.go.jp/library/open_innovation_hakusyo.html

[CR69] NEDO (2019) Annual report of the development of environmentally friendly process technology in Japan. https://www.nedo.go.jp/content/100905230.pdf

[CR70] Nikkei (2020) ArcelorMittal: steelmaking without coal, 5 trillion yen investment on natural gas and hydrogen. 2020.09.30. https://www.nikkei.com/article/DGKKZO64371720Z20C20A9TJ2000/

[CR71] Oda J, Akimoto K, Sano F, Tomoda T (2007). Diffusion of energy efficient technologies and CO_2_ emission reductions in iron and steel sector. Energy Econ.

[CR72] Oda J, Akimoto K (2019) Analysis of energy intensity of basic materials industry in Japan. In: local energy, global markets, 42nd IAEE international conference, May 29-June 1, 2019. International Association for Energy Economics.

[CR73] Oshiro K, Gi K, Fujimori S (2019). Mid-century emission pathways in Japan associated with the global 2°C goal: national and global models’ assessments based on carbon budgets. Clim Change.

[CR74] Peters GP, Andrew RM, Canadell JG, Fuss S, Jackson RB, Korsbakken JI (2017). Key indicators to track current progress and future ambition of the Paris Agreement. Nat Clim Change.

[CR75] Sakamoto S, Nagai Y, Sugiyama M et al (2021) End-use decarbonization and electrification: EMF 35 JMIP study. Sustain Sci.

[CR76] Scrivener KL, John VM, Gartner EM (2018). Eco-efficient cements: potential economically viable solutions for a low-CO_2_ cement-based materials industry. Cem Concr Res.

[CR77] Sugiyama M, Akashi O, Wada K, Kanudia A, Li J, Weyant J (2014). Energy efficiency potentials for global climate change mitigation. Clim Change.

[CR78] Sugiyama M, Fujimori S, Wada K (2019). Japan’s long-term climate mitigation policy: multi-model assessment and sectoral challenges. Energy.

[CR79] Sugiyama M, Fujimori S, Wada K, Oshiro K, Kato E, Komiyama R, Silva Herran D, Matsuo Y, Shiraki H, Ju Y (2021). EMF 35 JMIP study for Japan’slong-term climate and energy policy: scenario designs and key findings.

[CR80] UNIDO (United Nations Industrial Development Organization) (2016). Industrial development report 2016: the role of technology and innovation in inclusive and sustainable industrial development. UNIDO. https://www.unido.org/sites/default/files/2015-12/EBOOK_IDR2016_FULLREPORT_0.pdf

[CR81] UNIDO (2018). Industrial development report 2018. Demand for manufacturing: driving inclusive and sustainable industrial development. https://www.unido.org/resources-publications-flagship-publications-industrial-development-report-series/industrial-development-report-2018

[CR82] Van Sluisveld M, De Boer HS, Hof A, Van Vuuren D, Schneider C, Lechtenboehmer S (2019) Comparing quantitative industry decarbonization perspectives towards 2050 for Europe. IAMC 12th Annual Meeting, Tsukuba, Japan. https://www.iamconsortium.org/wp-content/uploads/2020/03/van_Sluisveld_NEW.pdf

[CR83] Vogl V, Åhman M, Nilsson LJ (2018). Assessment of hydrogen direct reduction for fossil-free steelmaking. J Clean Prod.

[CR84] Wakabayashi M, Arimura TH (2016). Voluntary agreements to encourage proactive fi rm action against climate change: an empirical study of industry associations’ voluntary action plans in Japan. J Clean Prod.

[CR85] World Steel Association (2019) World Steel in Figures 2019. https://www.worldsteel.org/en/dam/jcr:96d7a585-e6b2-4d63-b943-4cd9ab621a91/World%2520Steel%2520in%2520Figures%25202019.pdf.

[CR86] Yamaji K, Matsuhashi M, Nagata Y, Kaya Y (1991) An integrated system for CO_2_/ energy/GNP analysis: case studies on economic measures for CO_2_ reduction in Japan. Workshop on CO_2_ Reduction and Removal: Measures for the Next Century. 19–21 March 1991. International Institute for Applied Systems Analysis, Laxenburg, Austria.

