# Supplementary material for: Industrial decarbonization under Japan’s national mitigation scenarios: a multi-model analysis
Source: Sustain Sci. 2021 Feb 16;16(2):411–27. doi: 10.1007/s11625-021-00905-2 (PMC7970825; doi:10.1007/s11625-021-00905-2)
Supplement: Supplementary file 1 — Supplementary file1 (DOCX 1102 KB) [file 11625_2021_905_MOESM1_ESM.docx]

# Electronic supplementary material


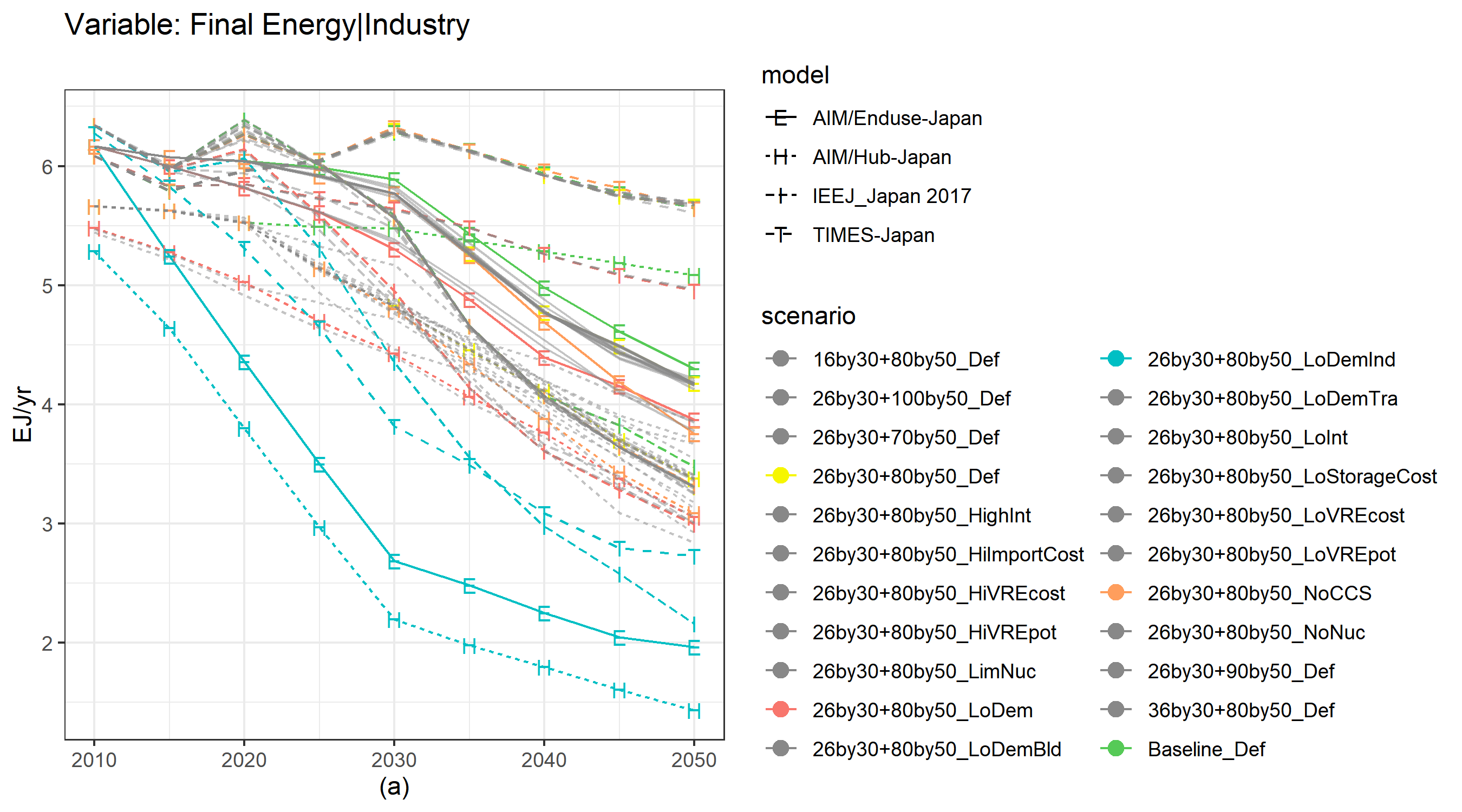


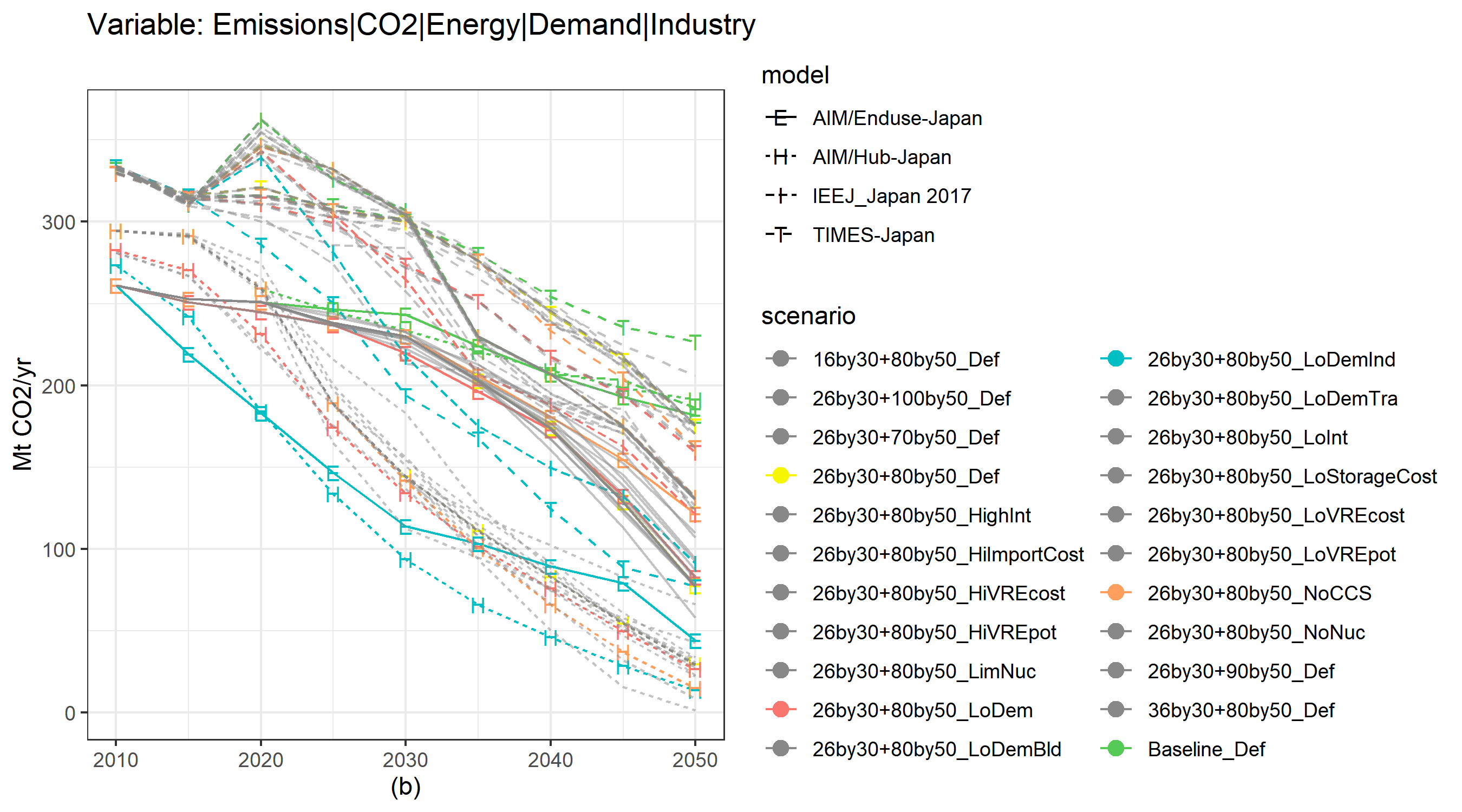


### Fig. ESM i Results in all EMF35 JMIP scenarios: Final Energy|Industry (a) and Emissions|CO2|Energy|Demand|Industry (b)


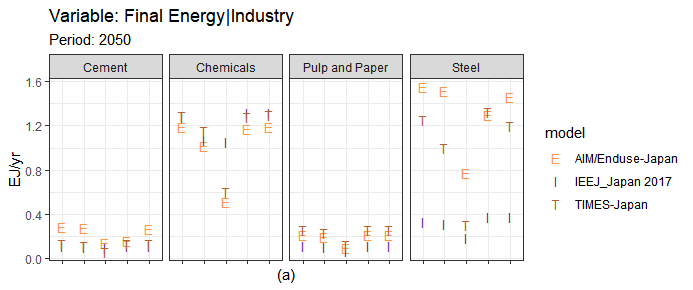


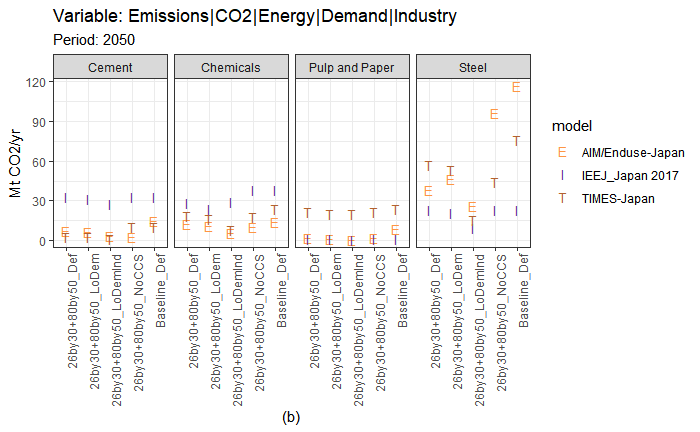


### Fig. ESM ii Results in industry sub-sectors’ final energy (a) and CO_2_ emissions (b) in 2050


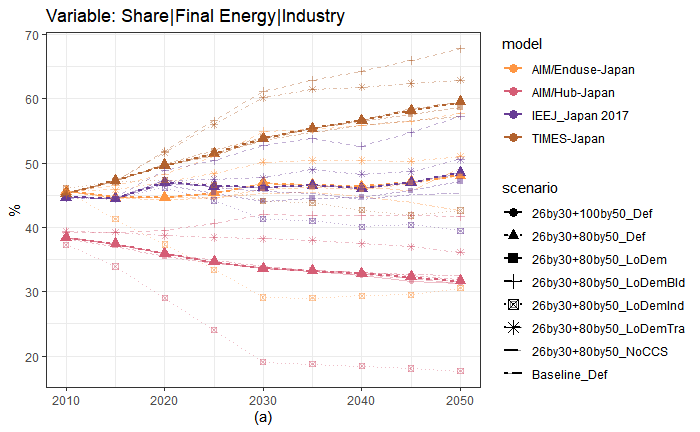


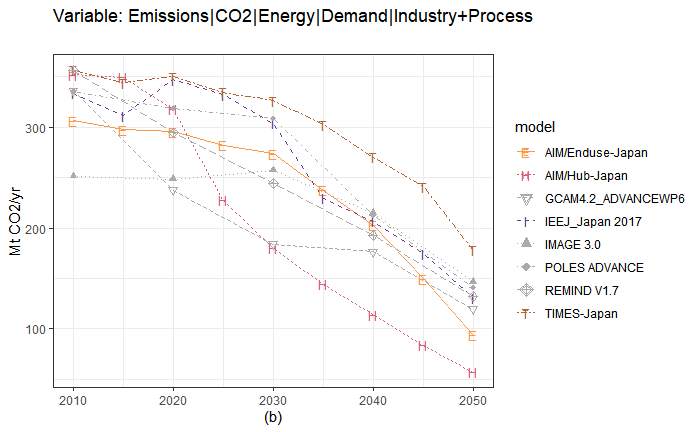


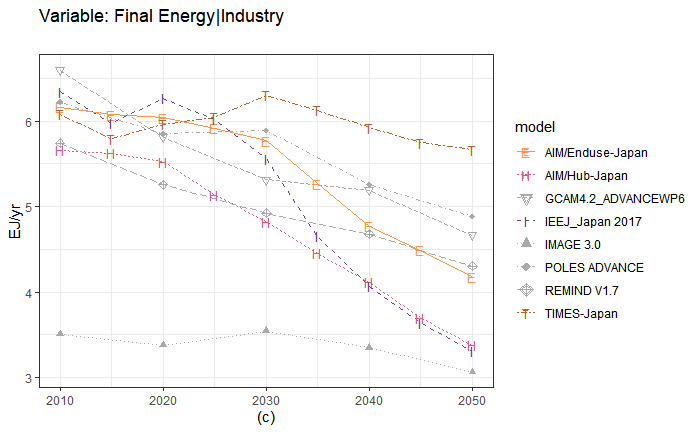


### Fig. ESM iii Industry’s share in final energy under other EMF35 JMIP scenarios (a), industrial emissions of more models (b), and industrial final energy of more models under selected scenario (c) under selected scenario

*Notes: Regarding the results from EMF35 JMIP, lines in (****b****) and (****c****) show the value under 26by30+80by50_Def. Regarding the results from model teams in countries/regions other than Japan, lines in (****b****) and (****c****) show the value under 2030_ Med2C (limit cumulative 2011-2100 CO2 emissions to 1600 GtCO2; more likely than not to stay below 2°C; implementing without strengthening until 2030). Source from ADVANCE Synthesis Scenario Database (version 1.0, Advanced Model Development and Validation for the Improved Analysis of Costs and Impacts of Mitigation Policies), project conducted during 2013-2016.*


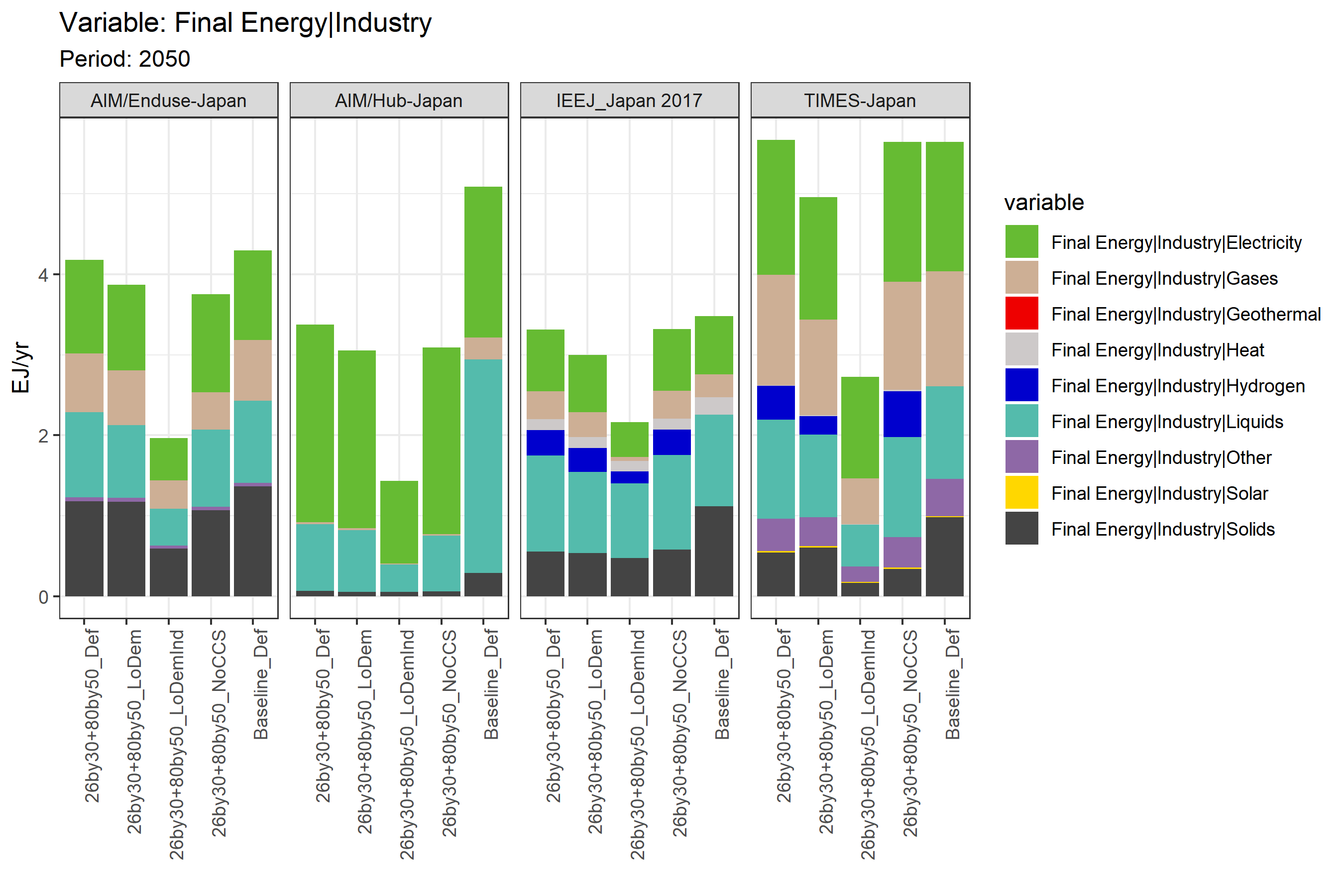


### Fig. ESM iv Decomposition of the Industry’s final energy by source in 2050


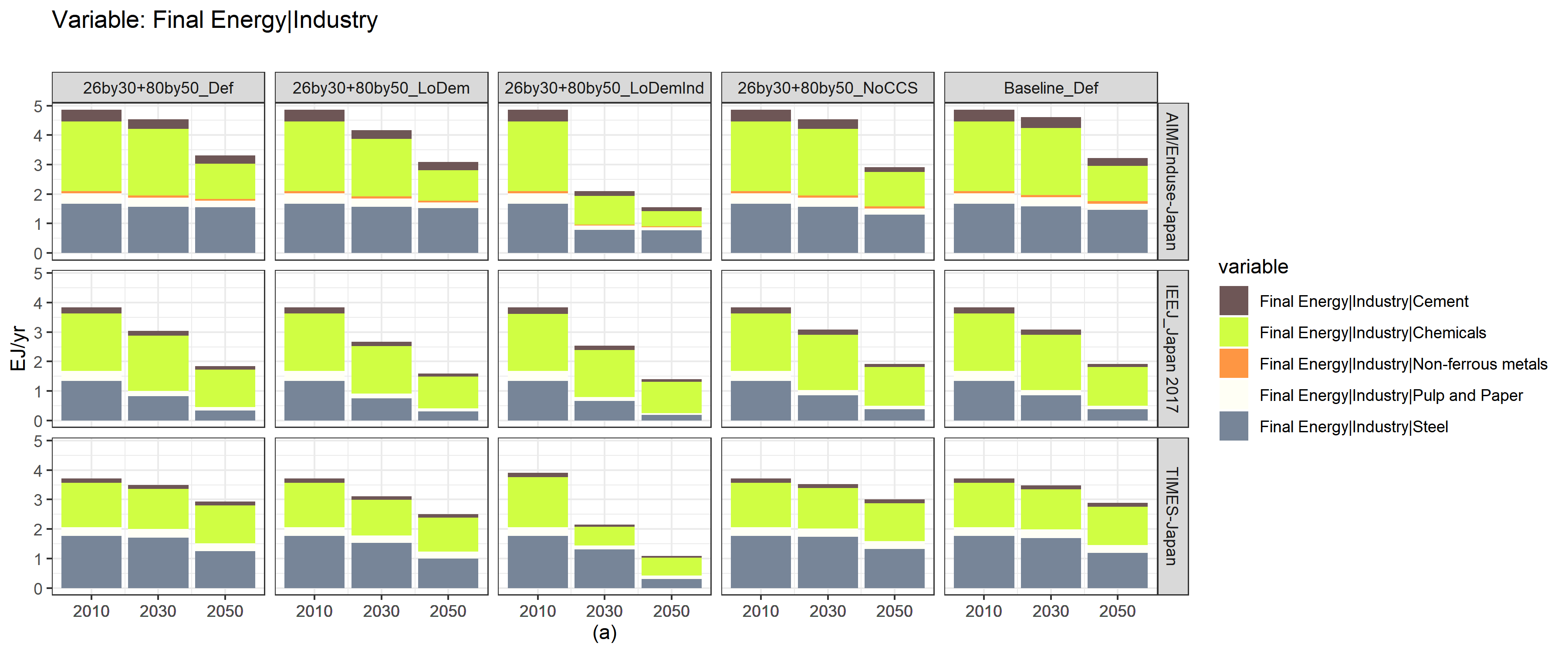


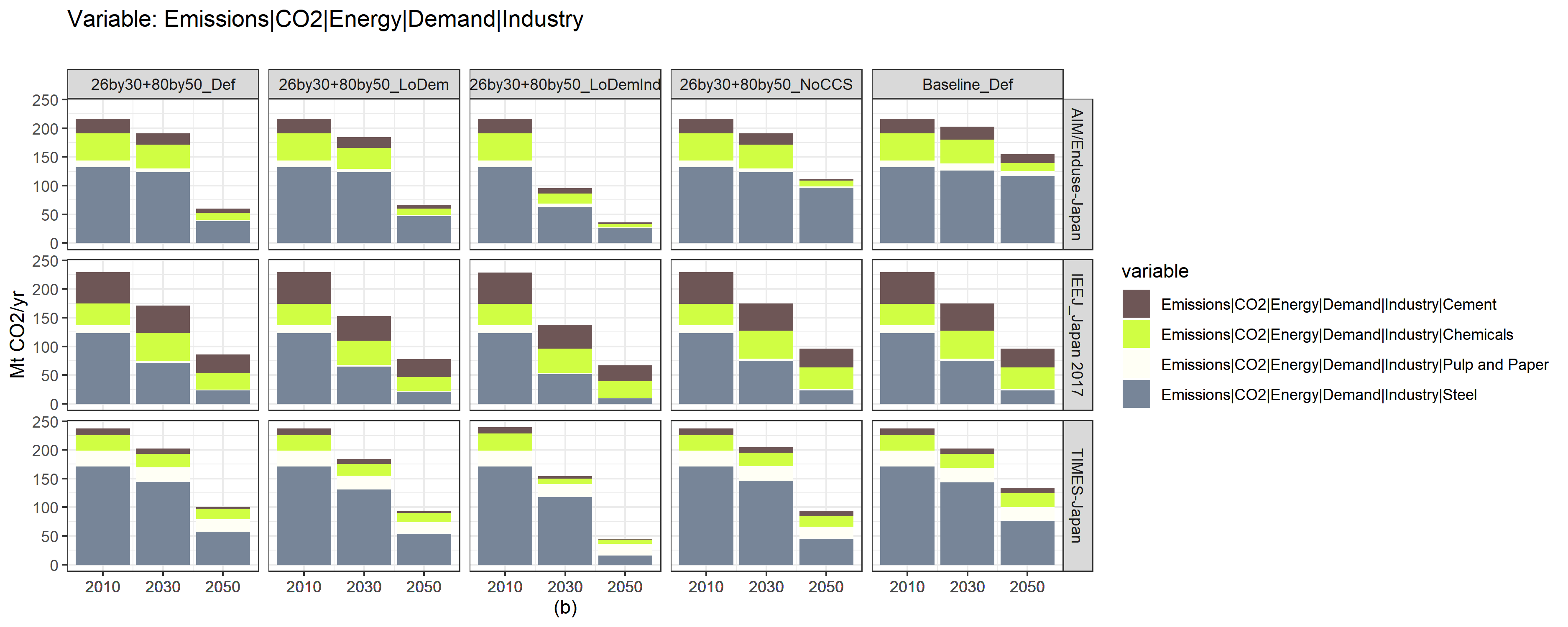


### Fig. ESM v Decomposition of the Industry’s final energy (a) and CO_2_ emissions (b) by sub-sector in 2050


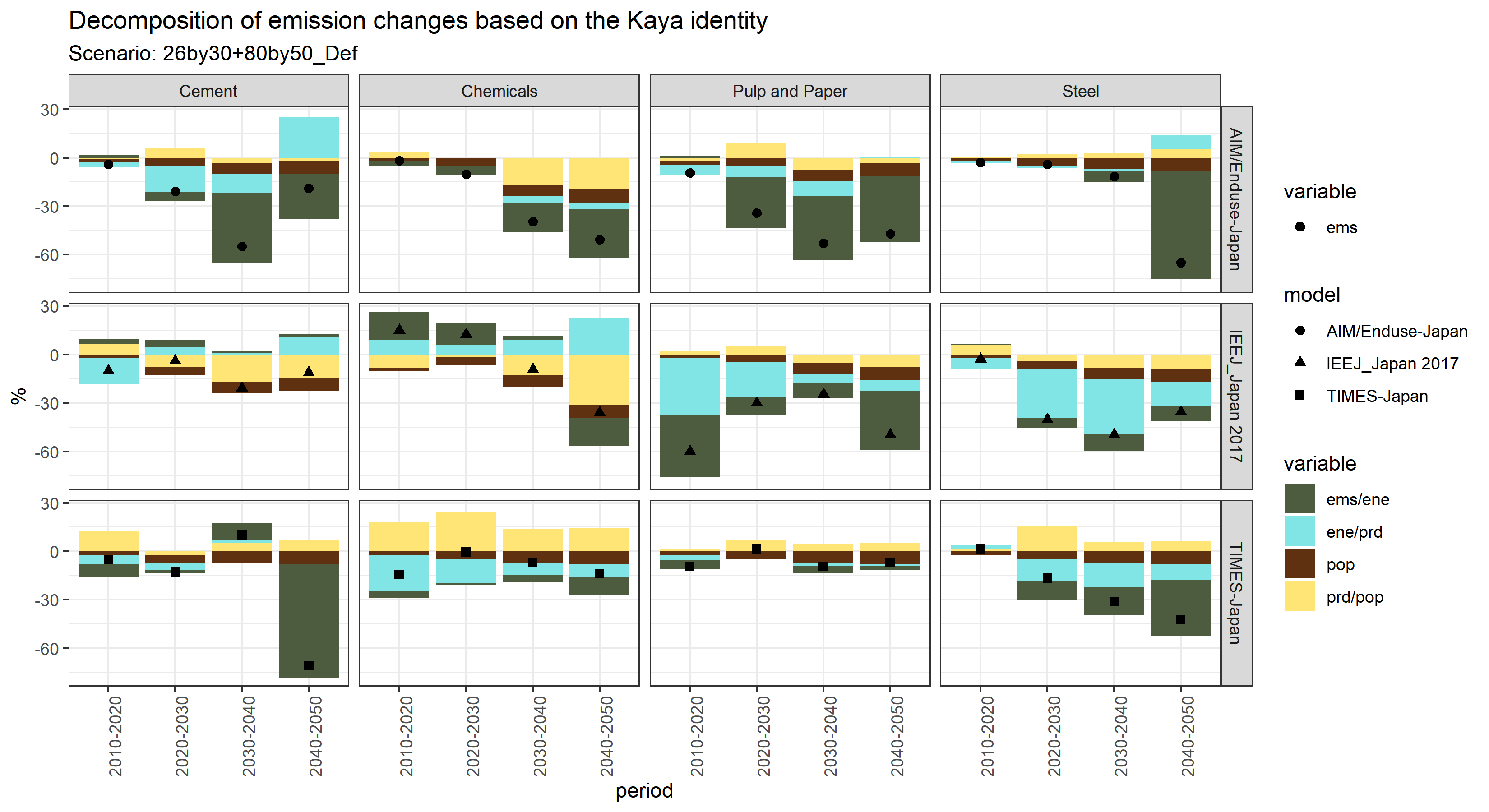


### Fig. ESM vi Decomposition of emission changes based on the Kaya identity


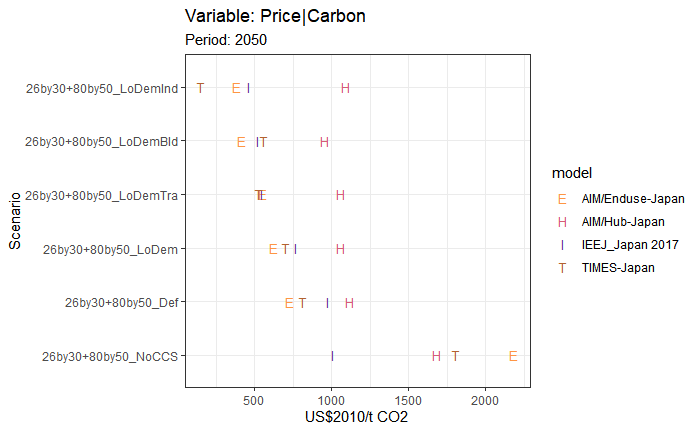


### Fig. ESM vii Carbon prices in 2050


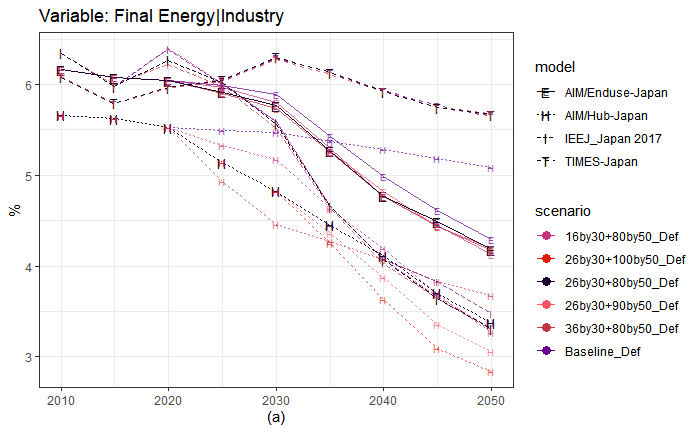


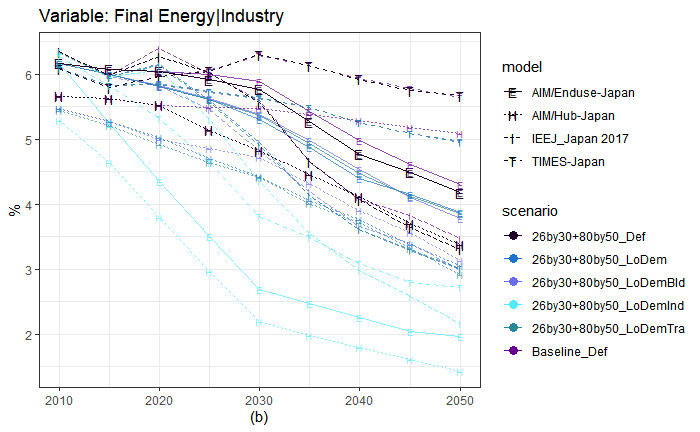


### Fig. ESM vii Sensitivity of variable Final Energy|Industry under policy scenarios (a) and demand scenarios (b)

*Notes: Policy scenarios include scenarios where Japan’s total CO2 emission will be reduced to 16-36 by 2030 and 80-100 by 2050. Demand scenarios include scenarios where the energy service demand are halved in total/building/industry/transportation sector.*

### Table ESM i Difference of industry energy/emission coverage among models

|  | AIM/Hub-Japan | AIM/Enduse-Japan | IEEJ_Japan | TIMES-Japan |
| --- | --- | --- | --- | --- |
| Industrial energy coverage | Industrial energy consumption (excludes coke oven gas and blast furnace gas, which are included in energy conversion sector). | Industrial energy consumption | Industrial energy consumption (excludes coke oven gas and blast furnace gas, which are included in energy conversion sector). | Industrial energy consumption |
| Data source of industrial energy | IEA | IEA | IEA | IEA |
| Industrial emissions | Industrial energy consumption and production processes | Industrial energy consumption and production processes | Industrial energy consumption only | Industrial energy consumption, production processes in cement sub-sector only |
| Data source of industrial emissions | EDGAR | National GHG Inventory Report | National GHG Inventory Report | National GHG Inventory Report |
